# Shape distortion in sintering results from nonhomogeneous temperature activating a long-range mass transport

**DOI:** 10.1038/s41467-023-38142-z

**Published:** 2023-05-09

**Authors:** Sandra M. Ritchie, Sasa Kovacevic, Prithviraj Deshmukh, Alexander D. Christodoulides, Jonathan A. Malen, Sinisa Dj. Mesarovic, Rahul P. Panat

**Affiliations:** 1 Department of Mechanical Engineering, Carnegie Mellon University, Pittsburgh, PA USA; 2 School of Mechanical and Materials Engineering, Washington State University, Pullman, WA USA

**Keywords:** Structural properties, Mechanical engineering

## Abstract

Sintering theory predicts no long-range mass transport or distortion for uniformly heated particles during particle coalescence. However, in sintering-based manufacturing processes, permanent part distortion is often observed. The driving forces and mechanisms leading to this phenomenon are not understood, and efforts to reduce distortion are largely limited to a trial-and-error approach. In this paper, we demonstrate that distortion during sintering results from mass-transport driven by nonhomogeneous temperature distribution. We then show that hitherto unknown mass transport mechanisms, working in the direction opposite to temperature gradient are the likely cause of distortion. The experimental setup, designed for this purpose, enables the quantification of distortion during sintering. Two possible mass transport mechanisms are defined, and the continuum model applicable to both is formulated. The model accurately predicts the transient and permanent distortion observed during experiments, including their size dependence. Methods to control distortion that can give rise to 4D printing are discussed.

## Abstract (web summary)

Shape distortion is a limitation for sintering-based manufacturing techniques, yet the fundamental causes are not fully understood. Here, the authors study and characterize such behavior and develop a continuum model to describe the effect and provide fundamental understanding to control distortion.

## Introduction

Several advanced manufacturing methods, such as additive manufacturing (colloquially called 3D printing), depend on particle sintering as one of the key process steps^1^. Sintering consolidates parts directly into the desired shapes with minimal additional processing, leading to low-cost near-net-shape manufacturing^2^. Unconstrained (freeform) sintering often results in parts that permanently deviate from the intended shape (i.e., they distort), while residual stresses develop if this deformation is constrained. The driving force and the mechanisms for this phenomenon are not well understood. As a result, the problem of shape distortion and residual stress in additive manufacturing is addressed via case-specific solutions obtained by trial-and-error^3–9^. While typically undesirable^8, 9^, such distortions, if controlled, could be used to manufacture complex shapes, or, in deployable systems, but such applications have not yet been reported. A mechanistic understanding of the permanent deformation caused by sintering is thus highly desirable.

Although shape distortion during sintering is often evident^5,7^, it has been difficult to quantify for the following reasons: (i) For typical part dimensions and shapes, sintering is done under constraints, limiting distortion and its measurements^10^, while producing residual stresses instead. (ii) Sintering for micron-sized particles takes place at relatively high temperatures (>500 °C) which makes in situ observations of shape change and temperature distributions difficult. Recent developments in 3D printing of nanoparticles^11^, enable us to overcome such experimental limitations. The resulting microscale nanoparticle structures sinter at relatively low temperatures (100–300 °C) and can be printed as freeform - unconstrained and statically determinate—structures. In such configurations, sintering shape distortion implies that either porosity gradients both exist and correspond to the observed final curvatures, or a long-range mass transport mechanism must operate. The existing sintering models^12–25^ do not account for such mass transport and thus cannot predict permanent distortion of freeform structures (temporarily less sintered/densified regions eventually catch up and erase any temporary distortions).

Here, we demonstrate and quantify permanent shape distortion in the absence of a corresponding porosity gradient, indicating the presence of long-range mass transport. We also define two possible mechanisms for the mass transport and formulate a continuum model applicable to both mechanisms. This model is then shown to accurately predict distortion behavior, and potential control mechanisms are discussed.

## Results and discussion

**Fig. 1:**
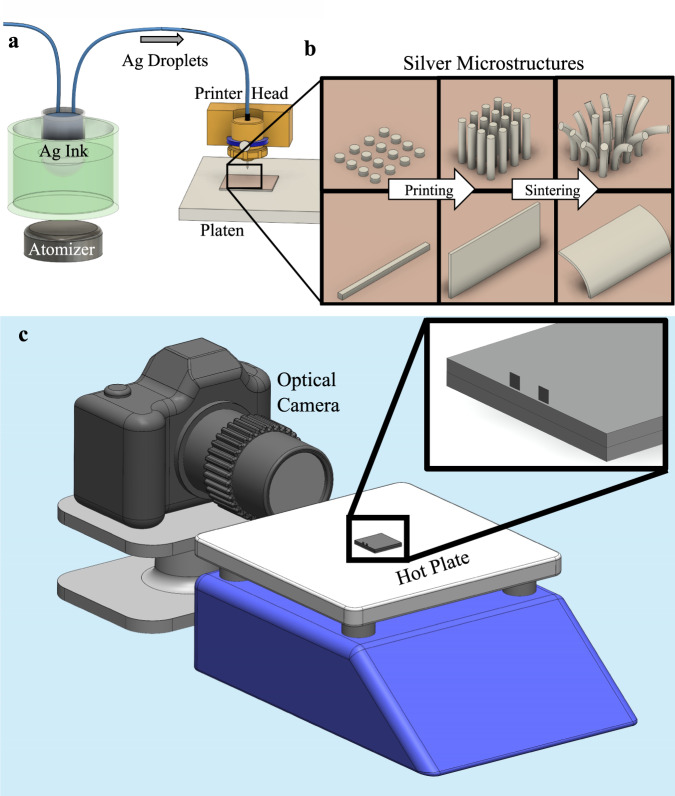
Aerosol Jet 3D printing experimental setup. **a** Ultrasonic atomizer creates microdroplets containing silver nanoparticles which are carried to the printer head and deposited on the substrate with a sheath gas to form three-dimensional structures. **b** AJ printed three-dimensional silver walls and pillars before (middle) and after (right) sintering. **c** Experimental setup used to sinter the freestanding structures in **b** on a hot plate with an optical camera recording their shape deformation in real-time.

To measure the shape distortion during sintering, minimally constrained three-dimensional micro and mesoscale structures of nanoparticles were constructed using Aerosol Jet (AJ) 3D printing. An ultrasonic atomizer (Fig. 1a) was used to create an aerosol of solvent droplets containing silver nanoparticles. The aerosol was then transported to the printhead using N_2_ gas. The droplets were focused onto a heated platen using a sheath gas (also N_2_) to create the nanoparticle structures with a minimum feature size of 10 µm. A computer-aided design (CAD) program controlled the shape of the 3Dprinted structures. Two types of structures were considered: micropillars and microwalls, with wall thicknesses in the range of tens to a hundred micrometers. Sintering was achieved by heating the structures on a hot plate while recording their curvature using an optical camera. Details of the AJ 3D printing process are provided in the Methods section.

Schematics of the freestanding 3D printed micropillar and microwall structures of nanoparticles are shown in Fig. 1b and remain stable during printing on a heated platen^11^. Initially, the heat from the platen removes the solvents from the ink droplets to create a solidified green structure containing the metal nanoparticles. As the next ink droplet arrives, the surface force is high enough (compared to inertial force) so that the droplet sticks to the green structure^11^. The micropillars and microwalls in this state consist of unsintered silver nanoparticles held together by the binder. The structures are then sintered on a hot plate, where curvature is captured by the optical camera (Fig. 1c).

**Fig. 2:**
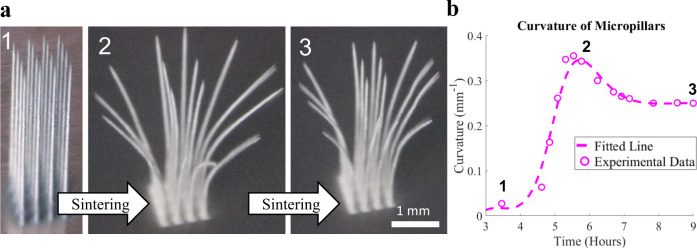
Sintering distortion of micropillars. **a** Flower-like opening of an AJ printed 4×4 array of silver micropillars, each with 60 μm diameter and 4 mm height, heated to 300 °C over 12 hours. The three images show snapshots at the initial stage, after distortion, and after a partial recoil. A video of this curvature evolution is shown in Supplementary Movie 1. **b** Average curvature of the outermost micropillars shown in **a** as a function of time during heating. The formation of peak curvature and recoil are clearly observed. The dotted line represents a fit to the experimental data.

**Fig. 3:**
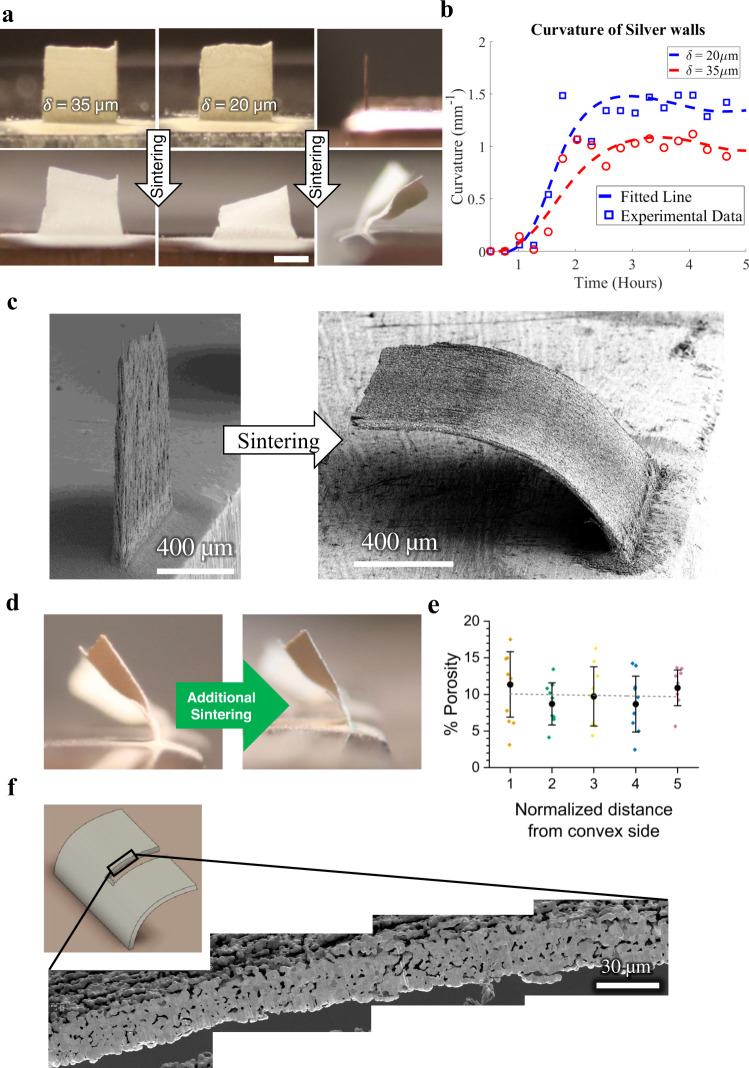
Experimental evolution of microwall curvature during sintering. **a** Bending of AJ printed 20 and 35 μm thick nanoparticle walls (1.5 × 1.5 mm) during sintering. The front and side views clearly demonstrate measurable permanent deformation. Scale bar is 0.75 mm. **b** Curvature of the walls shown in **a** as a function of time indicating the peak and partial recoil. **c** Representative SEM image of a 35 μm thick wall before and after sintering and curvature formation. **d** A bent nanoparticle wall that showed no additional shape change upon further sintering after the main experiment. **e** Porosity measurements for the wall shown in **d** through the thickness. No significant porosity gradient observed in these measurements (i.e., there is no statistical variation in porosity which could support the curvature seen). Error bars represent standard deviation. **f** A representative cross-section of the bent nanoparticle wall in **d**. Many such images were used to compile the graph in **e**.

An AJ 3D printed 4 × 4 micropillar array is shown in Fig. 2a. The as-printed micropillars have a diameter of 60 µm and a height of 4 mm (aspect ratio of 1:67). The micropillars are attached only at the substrate, making the structure statically determinate. The micropillar array was sintered on the hot plate (for details, see the Methods section). The process is also shown in a timelapse Supplementary Movie 1, where the sintering front (bright color) is clearly observed along with the formation of the curvature for the micropillars. The average curvature of the outer micropillars in the array as a function of time during sintering is plotted in Fig. 2b (measurement details are given in the Methods section). Starting from no curvature in the as-printed state (position 1), the curvature increases and reaches the maximum (position 2). This is followed by a limited recovery until the final, permanent curvature is obtained (position 3). Although the results shown in Fig. 2 are noteworthy and provide valuable information, the micropillars can bend in arbitrary directions, making accurate curvature measurements difficult. To address this issue, we created a simple 1D microwall geometry (Fig. 3), which bends in a predictable direction so that the curvature can be accurately measured via optical images. We tested wall thicknesses in the range of 20–140 μm, each with a length and height of 1.5 mm. Details of the curvature measurements of the microwalls during heating of the platen and sintering are given in the Methods section. The optical images of microwalls, as-printed and after sintering, with 20 μm and 35 μm wall thickness are shown in Fig. 3a, and the evolution of their curvatures during sintering in Fig. 3b. As in the case of micropillars, the curvature of the microwalls increased as the sintering proceeded to reach a peak value, then exhibited a mild recovery. Representative videos of bending of 20 μm and 35 μm thick AJ 3D printed microwalls upon heating are shown in Supplementary Movies 2 and 3. The SEM image of a 35 µm thick microwall after sintering is shown in Fig. 3c. Note that factors such as the moving boundaries of the microwall (and hence the dynamically changing shape factor for the structure) and dynamically changing material structure (3D printed green structure to the sintered silver wall) and emissivity during bending precluded us from accurately measuring the temperatures on the two sides of the microwall. Nevertheless, while the thermal camera measurements do not provide accurate temperature, they do indicate the qualitative differences in temperature, and our measurements yielded one consistent result: the microwalls always bend toward the hotter side.

The permanent curvature of the microwalls suggests that one of the following statements must be true: either the final product has higher porosity at the convex side, or the mass has been transferred from the concave to the convex side. To resolve this dilemma, we cross-sectioned bent microwalls using the focused ion beam (FIB) technique. The details of this measurement are given in the Methods section, and additional data and analysis are shown in Supplementary Fig. 1 and Supplementary Note 1. The porosity as a function of the distance from the convex side of the wall was characterized for three curved walls from the experiments in this work. No statistical bias was observed, but the data showed high scatter (see Supplementary Fig. 1a–c) due to large pore sizes. To address this issue, a bent sample was re-sintered after the initial experiment to a higher temperature of 400 °C for an extended period of time (10 hr) to further reduce porosity^26^. Indeed, the re-sintered wall showed lower average porosity of 9.9%. The wall exhibited no additional change in shape upon further sintering (see Fig. 3d). This in itself shows that curvature is permanent and cannot be removed by further sintering to ensure uniform density of the structures. The porosity data (average and standard deviation of nine different sections along the length of the wall), given in Fig. 3e, indicates no significant difference in porosity through the wall thickness between the concave (shorter) and the convex (longer) sides of the structure. A representative SEM image of the FIB cut cross-sections across the microwall thickness is shown in Fig. 3f. The measured curvature for all cross-sectioned walls was also compared to the curvature predicted by the observed porosity (Supplementary Fig. 1e with details in Supplementary Note 1), and this analysis showed no correlation between these values. In the absence of a meaningful correlation in porosity and curvature, it is unlikely that differential porosity could explain the bending of the AJ printed walls. We conclude that curvature is much more likely the result of a mass transport mechanism operating in the direction normal to the wall midplane, and opposite to the temperature gradient, i.e., the mass moves from hot to cold regions.

**Fig. 4:**
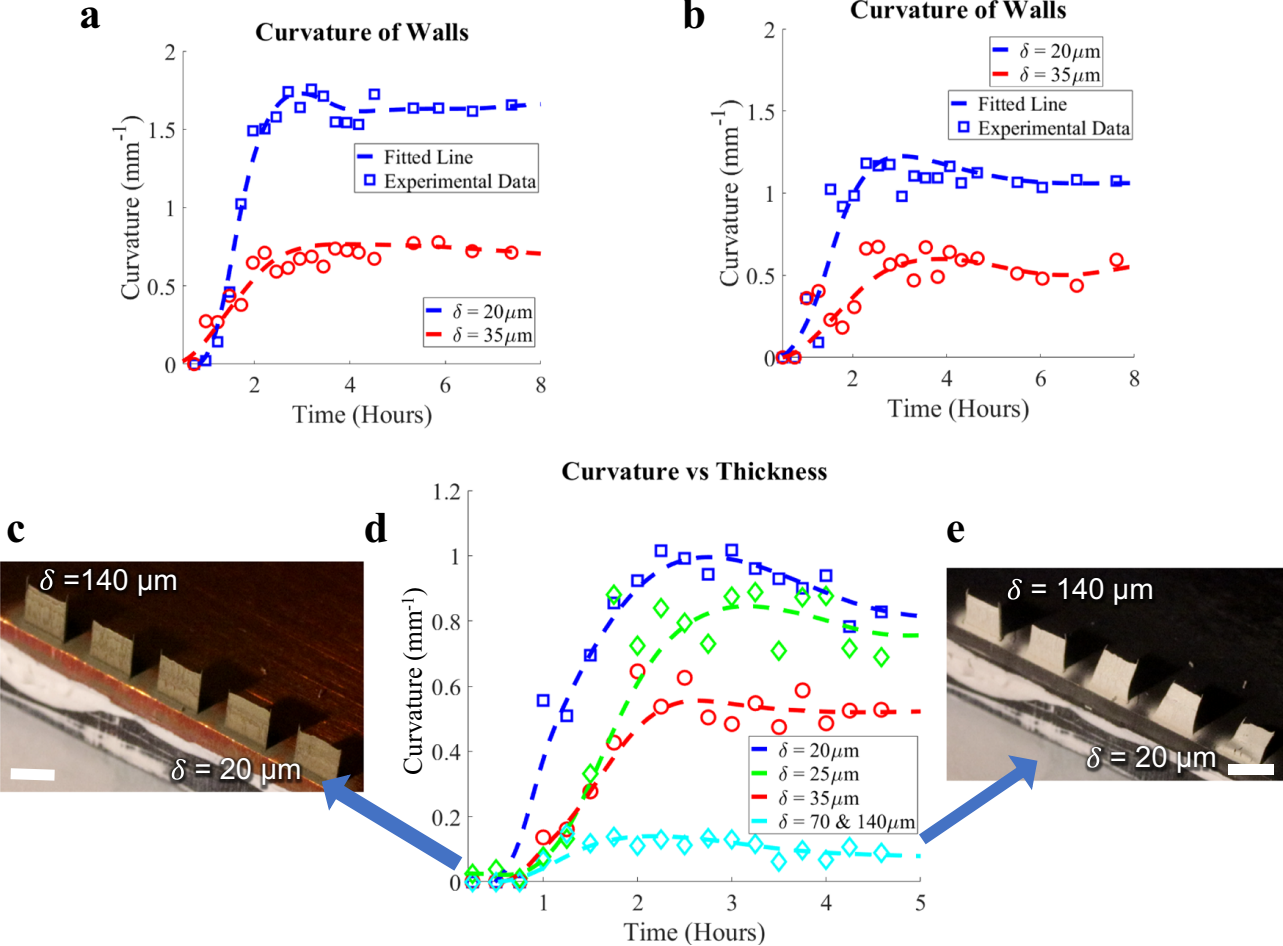
Experimental data and curve fits showing decreasing curvature with increasing thickness (*δ*) of the nanoparticle walls during sintering. **a**, **b** Additional curvature data for 20 μm and 35 μm thick silver nanoparticle walls after sintering at 300 °C. **c** AJ printed nanoparticle walls (1.5 × 1.5 mm) with increasing thickness from 20 μm to 140 μm. Scale bar (1.5 mm). A video showing the bending during sintering is included in Supplementary Movie 4. **d** Curvature of the walls shown in **c** as a function of time indicating the peak curvature and partial recoil. **e** Final deformation state of the silver nanoparticle walls after sintering at 300 °C. Scale bar is 1.5 mm.

We have established repeatability of our experiments with twelve total sintering tests involving six 20 μm and six 35 μm thick microwalls. The curvature data for these experiments as a function of the heating time are shown in Fig. 4a, b and Supplementary Fig. 2a–2d (also see Supplementary Table 1), which indicates trends similar to that observed in Fig. 3a, b. The effect of microwall thickness was studied via three more experiments, each having five microwalls with thicknesses ranging from 20–140 μm. Although the evolution of curvature for each microwall followed the same trends during sintering, the thicker walls bent significantly less than the thinner walls (Fig. 4c–e and Supplementary Fig. 3a, b). For example, the peak curvature of the 20 μm wall was about 1 mm^−1^, while that of the 35 μm wall was ~0.5 mm^−1^. For all experiments, the 70 μm and 140 μm walls exhibited low curvature throughout the sintering process.

The reality of mass transport thus being established, we turn to the questions of driving force and the underlying mechanism of the mass transport. Based on the consistency of walls bending towards the hotter side, we hypothesize that: (1) temperature gradient causes sintering rate gradient, (2) The sintering rate gradient causes mass transport toward less sintered regions. We consider these hypotheses in detail below.

**Fig. 5:**
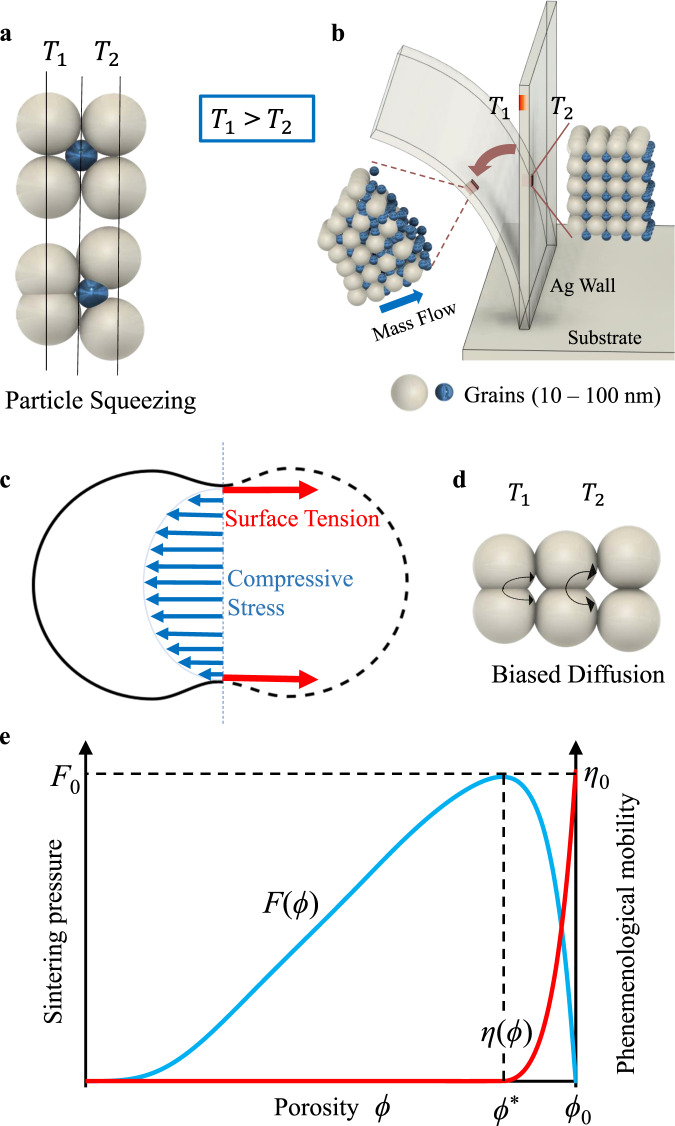
Two physical mechanisms of mass transport and elements of the common macroscopic model. Particle squeezing mechanisms **a** on the particle scale and **b** on a larger scale. **c** Typical micro-stress distribution for a pair of particles. **d** Biased diffusion mechanisms. **e** Phenomenological mass transport mobility for particle squeezing mechanism and the sintering pressure as functions of porosity.

(1) Owing to the high thermal conductivity of silver, a high and permanent temperature difference across the wall thickness in the absence of internal heat sources is not possible. We note that most of the bending occurs in the early stages of sintering when the sintering front can be observed moving up from the heated base (see the color change accompanying the formation of curvature in Supplementary Movie 4). Therefore, our thermal analysis focuses on this time interval. We note that the thermal processes in the system likely occur non-homogeneously in this time interval. These processes include binder burnout and the exothermic nature of sintering and can give rise to transient temperature gradients across the wall thickness. We measured the thermal processes via thermogravimetric analysis (TGA) and dynamic scanning calorimetry (DSC), see Supplementary Fig. 4 (also see Methods section). The main results of these experiments are: (i) solvent evaporation likely occurred during the printing process and (ii) both electrical and thermal conductivity show two large increases during the initial heating phases, likely due to initial binder removal and the onset of sintering before and during the beginning of bending, respectively. The experimental thermal analysis is discussed in Supplementary Note 2. The non-uniform thermal processes can give rise to transient temperature gradients based on the thermal conductivity of the structure, which was also calculated experimentally in Supplementary Fig. 5 (see Supplementary Note 2 for details). The details of the thermal simulations are shown in Supplementary Note 3 and Supplementary Fig. 6. The simulations show that: (i) without mass transport, any developed curvature is transient and fully reversed by further sintering, and, (ii) upon localized perturbation, the exothermic nature of sintering produces a transient thermal instability as the sintering front moves up, producing a temporary temperature difference through the wall thickness of 3-7 °C (depending upon the wall thickness).

(2) Two mass transport mechanisms are possible: particle squeezing and biased diffusion. Once the solvent is burned out, the unsintered sample is essentially a granular material: near-spherical particles of varying sizes with the remaining binder in a disordered assembly. As graphical representations of disordered assemblies are not explanatory, we will use grossly simplified periodic assemblies (Fig. 5) to explain the two mechanisms. To understand the particle squeezing mechanism, consider the initially periodic array of larger and smaller particles, as illustrated in Fig. 5a. The temperature gradient is horizontal. The vertical line of particles at a higher temperature shortens more than the colder one and squeezes the smaller particles toward the colder side. This displacement is expected to be small and local. However, if one imagines a sequence of such cells in the direction of temperature gradient, the many small mass displacements add up to a significant long-range irreversible mass displacement towards the colder side, as illustrated in Fig. 5b.

The biased diffusion mechanism has been considered in the literature on rapid (spark-plasma) sintering of ceramics^27–31^, but only as directly driven by the temperature gradient (Schottky^32^ vacancy diffusion mechanism). We show in Supplementary Note 4 that in our case (silver nanoparticles), the vacancy diffusion flux resulting from the temperature gradient is several orders of magnitude smaller than the flux resulting from the contact stress gradient and is, therefore, negligible. The sketch of micro-stress distribution across the neck between two partially sintered (initially spherical) particles is shown in Fig. 5c. The high neck curvature and compressive micro-stresses in the center of the neck drive the surface and bulk diffusion of atoms towards the neck surface. Micro-stresses and surface tension are equilibrated and produce no macro-stress. In homogeneous sintering, the ideal necks are symmetric, so no side is preferred to receive diffusion atoms. However, under temperature gradient, the biased diffusion occurs (Fig. 5d), directed towards higher curvature necks, i.e., towards lower temperature. We note that the temperatures are high enough to provide high surface mobility of atoms. Even if such mass transport is confined to the neighboring necks only, in a (quasi-) periodic array stretching along the temperature gradient, the small mass displacements add up to a significant total mass displacement.

Although at this stage the question of operating mechanism is open, the thermodynamic forces that drive both mechanisms are the same. They arise from the tendency of the system to minimize the micro-elastic energy corresponding to the micro-stresses. The common macroscopic thermodynamics in the two mass transport mechanisms enable us to formulate the macroscopic continuum theory for nonhomogeneous sintering (in the absence of macro-stress) that accounts for mass transport.

Consider first homogeneous freeform sintering. In the absence of mass transport and constraints, the only strain during the sintering is the sintering (volumetric) strain ${\theta }_{S}$, directly related to the porosity $\phi$:

1 $${\theta }_{S}=\phi -{\phi }_{0}\le 0{{{{{\rm{;}}}}}}\,{\dot{\theta }}_{S}=\frac{d{\theta }_{S}}{{dt}}=\dot{\phi },$$

where ${\phi }_{0}$ is the initial porosity. When the mass transport is present, the additional mass transport (volumetric) strain ${\theta }_{m}$ appears, subject to mass conservation law: ${\dot{\theta }}_{m}=-\nabla \cdot {{{{{\bf{q}}}}}}$. The flux, ${{{{{\bf{q}}}}}}$, is the solid volume flux at constant porosity. If the elastic and thermal compressibility of solid is neglected, this is equivalent to the mass flux. The total volumetric strain is then:

2 $$\theta={\theta }_{S}+{\theta }_{m}.$$

The sintering is driven by the minimization of the surface energy of pores. The surface energy density $P(\phi )$ (surface energy per unit volume) is a function of porosity and satisfies the following constraints:

3 $$P\left(\phi \right)\ge 0{{{{{\rm{;}}}}}}{P}\left(0\right)=0{{{{{\rm{;}}}}}}\,\frac{{dP}}{d{{{{{\rm{\phi }}}}}}}=F(\phi )\ge 0$$

The dependence of the sintering pressure $F(\phi )$ on the porosity is shown in Fig. 5e. Starting from the initial porosity ${\phi }_{0}$, the sintering pressure rapidly reaches a peak as porosity decreases and is diminished at low porosities. To define the thermodynamic driving force for the mass transport, we imagine fictitious constraints preventing such transport. These result in internal (self-equilibrating) forces and internal strain energy (even in the absence of external loads and macro-stresses). Thus, the produced micro-elastic strain energy increases with increasing sintering strain gradient and is relaxed by the mass transport strain gradient. Upon inspection (e.g., Fig. 5b), it is clear that these gradients must point in the same direction to cancel out. The simplest model for such internal micro-elastic strain energy density is the quadratic form of the two gradients:

4 $$Q\left(\nabla {\theta }_{S},\nabla {\theta }_{m}\right)=\frac{1}{2}B{l}^{2}{(\nabla {\theta }_{S}-\alpha \nabla {\theta }_{m})}^{2}\ge 0,$$

where $B$ has the dimensions of elastic modulus, $l$ is the characteristic length (e.g., average particle size), and the coefficient $\alpha$ represents the ratio of sintering strain gradient and the mass transport strain gradient which fully relaxes the micro-elastic energy. It is expected to be of order 1. In our simulations, we assume $\alpha=1$. We consider the macro-stress-free problem with an externally controlled temperature field. The changes in temperature resulting from dissipation are negligible compared to the imposed temperature, so that heat conduction is not modeled. We assume that the temperature affects mobilities but does not affect the potential energy of the system. The potential energy of the system thus includes the surface energy density $P(\phi )$ and internal micro-elastic strain energy density $Q\left(\nabla {\theta }_{S},\nabla {\theta }_{m}\right)$:

5 $$E={\int }_{V}\left\{P\left(\phi \right)+Q\left(\nabla {\theta }_{S},\nabla {\theta }_{m}\right)\right\}{dV}.$$

The governing equations (derived in the Methods section below) for the domain $V$, have the form:

6 $$\left\{\begin{array}{c}\dot{\phi }=-\mu (T)\left[F\left(\phi \right)-B{l}^{2}\left({\nabla }^{2}\phi -\alpha {\nabla }^{2}{\theta }_{m}\right)\right] \hfill \\ {\dot{\theta }}_{m}=-\eta (\phi )\alpha B{l}^{2}({\nabla }^{2}\phi -\alpha {\nabla }^{2}{\theta }_{m})\hfill \end{array}\right\}\,{{{{{\rm{in\ }}}}}}{V},$$

while the boundary conditions on the boundary $\partial V$ with unit outer normal ${{{{{\bf{n}}}}}}$, are

7 $${{{{\bf{n}}}}}\cdot \nabla {\theta }_{S}=\alpha {{{{\bf{n}}}}}\cdot \nabla {\theta }_{m}\,{{{\rm{on}}}}\,\partial V.$$

The phenomenological mass mobility coefficient $\eta (\phi )$ is illustrated in Fig. 5e for the particle squeezing mechanism. It describes the available space in the network for particle displacement, but also the state of sintering necks, i.e., the availability of particles for displacement. Consequently, it has positive values only in the early stages of sintering. It has the maximum value initially $\eta \left({\phi }_{0}\right)={\eta }_{0}$, then rapidly decreases to zero $\eta (\phi < {\phi }^{*})=0$. Below the critical porosity for ${\phi }^{*}$, the particle network is frozen and the particle squeezing mechanism cannot operate. We expect that $\eta (\phi )$ for the biased diffusion mechanisms will decrease more gradually towards zero.

For homogeneous sintering (uniform temperature and porosity at all times), the sintering rate takes the form:

8 $${\dot{\phi }}_{{homo}}=-\mu \left(T\right)F\left(\phi \right).$$

This function can be extracted from homogeneous sintering experiments. The sintering mobility $\mu \left(T\right)$ is expected to have the standard form of thermally activated processes:

9 $$\mu=\nu {{\exp }}\,\left\{-\frac{\Delta G}{{kT}}\right\}.$$

where $\Delta G$ is the activation energy of the relevant diffusion process (bulk or surface), $k$ is the Boltzmann constant, and $\nu$ is the preexponential related to the grain size and the attempt frequency in elementary diffusion steps. The mobility here is the effective mobility for a grain, not the diffusion mobility. It will scale with the grain size in a manner similar to creep strain rate^33^.

We consider a slender freestanding wall of uniform thickness $\delta$ with a temporary lateral temperature gradient $\Delta T/\delta$. The sintering is done under nominal temperature ${T}_{0}\gg \Delta T$. The resulting one-dimensional problem is solved numerically (details in Supplementary Note 5) for various thicknesses of freestanding walls and $\Delta T$ estimated from numerical simulations of Supplementary Note 3. Dimensional analysis (given in the Methods Section) indicates that the problem is governed by three non-dimensional parameters:

10 $$\bar{\alpha }=\frac{\alpha {\eta }_{0}}{{\mu }_{0}}{{{{{\rm{;}}}}}}\,\beta=\frac{B}{{F}_{0}}{\left(\frac{l}{\delta }\right)}^{2}{{{{{\rm{;}}}}}}\,\Delta \bar{T}=\frac{\Delta T}{{T}_{0}},$$

where ${\eta }_{0}$ and ${F}_{0}$ are shown in Fig. 5e, and ${\mu }_{0}=\mu ({T}_{0})$ is given in Eq. (9). In our experiments $\delta$ is controlled, while $\Delta T$ and ${T}_{0}$ are estimated from simulations (Supplementary Note 3). We use the same nanoparticles, binder, and printing method in all experiments. Therefore, the parameters $\bar{\alpha }$ and $\beta {\delta }^{2}=B{l}^{2}/{F}_{0}$ are expected to be the same for all tests. We select $\bar{\alpha }$ and $\beta {\delta }^{2}$ for the overall best fit to all experiments, then test the consistency of computational and experimental results with respect to the variations in the wall thickness.

**Fig. 6:**
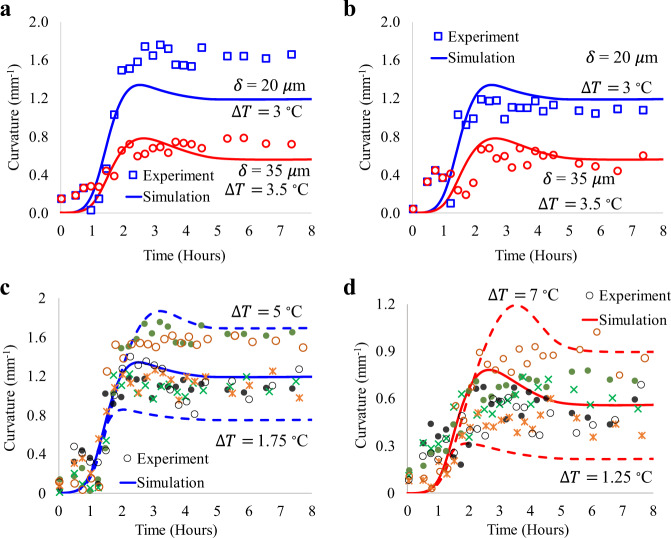
Comparison between experimental and computational results. Temperature difference for 20 μm and 35 μm walls is 3 °C and 3.5 °C, respectively, based on the heat transfer analysis in Supplementary Note 3. The model parameters are given in Supplementary Table 1. **a** Curvature of the 20 and 35 μm walls as a function of time for the first experimental group. **b** Curvature of the 20 and 35 μm walls as a function of time for the second experimental group. **c** Sensitivity of curvature with respect to the temperature difference for all six experimental groups for 20 μm walls. The solid blue line is obtained using the temperature difference of 3 °C. **d** Sensitivity of curvature with respect to the temperature difference for all six experimental groups for 35 μm walls. The solid red line is obtained using the temperature difference of 3.5 °C.

A comparison between the simulations and experiments is given in Fig. 6, with additional results provided in Supplementary Fig. 2a–d. The computational model predicts peak and permanent curvatures during nonhomogeneous sintering observed experimentally with reasonable accuracy. Since the temperature difference obtained from the thermal perturbations in Supplementary Note 3 is an estimation, we also carried out the sensitivity analysis for the 20 μm and 35 µm thick walls with respect to temperature difference. The sensitivity of curvature to the temperature difference for all six experimental groups for 20 μm and 35 μm thick microwalls is shown in Fig. 6c, d, respectively. The experimental data falls within a reasonable range of values.

We also carry out a parametric analysis by varying model parameters (details provided in Supplementary Note 5) and two results bear emphasis:

1. The increasing effective-stiffness-to-sintering-pressure ratio $B/{F}_{0}$ produces higher permanent curvature but diminishes the transient curvature, i.e., the difference between the peak and permanent curvatures (Supplementary Fig. 7).
2. The effect of the particle size $l$ cannot be assessed based on the continuum model without more precise knowledge of the operating mass transport mechanisms. Hidden in the continuum model is the effect of particle size on: the maximum sintering pressure ${F}_{0}$, the characteristic sintering mobility ${\mu }_{0}$, and the characteristic mass transport mobility ${\eta }_{0}$. Thus, the question of particle size effect remains open.

We thus show that freestanding structures of unsintered nanoparticles fabricated via Aerosol jet 3D printing exhibit distortion during sintering when heated on a hot plate. The experimental setup allows in situ measurements of curvatures during heating. The absence of detectable porosity gradients in the final structure indicates the existence of a mass transport mechanism, moving the mass towards lower temperature regions. The two candidate physical mechanisms are driven by the same physical phenomenon—the relaxation of micro-elastic strain energy—and are thus describable by one macroscopic continuum model. The resulting model captures the trends in the development of the curvature of the freestanding structures with reasonable accuracy.

Finally, there is the tantalizing possibility of using predictable, controlled distortion for creating complex curved shapes in the sintering phase of 3D printing. For that, a full understanding of the mass transport is needed as well as the effective means of controlling temperature differences, including determining the potential for binders in the ink to provide lubrication for particle motion. Nanoparticles are already printed in 3D shapes, such as microlattices and spirals^11^. Appropriate inverse programming can lead to previously impossible microscale shapes/structures and expand possibilities in the field of 4D printing.

## Methods

### Sample fabrication

We considered two geometries, namely, micropillar arrays and freestanding microwalls to study the shape distortion during sintering. These freestanding, unconstrained structures are fabricated using an Aerosol Jet 3D printer (Model AJ-300, Optomec, Inc., Albuquerque, NM) as shown in Fig. 1a. Silver nanoparticle ink used to construct the micropillars (Prelect TPS 50 G2, Clariant Group, Frankfurt, Germany) had particles sizes in the range of 30–50 nm. The silver nanoparticle ink (i.e., dispersion) used to construct the microwalls (Metalon JS-A221AE, Novacentrix, Austin TX) had an average particle size of 47 nm. The silver inks were diluted with deionized water with a ratio of 3:1. Prior to printing, the geometry of the structures was drawn in AutoCAD using a program in the software AutoLISP (AutoCAD 2015, Autodesk Inc., San Rafael, CA) and converted to a prg file which can be read by the printer software. The 150 μm diameter nozzle was used to fabricate all the structures. Ultrasonic atomization was used in the AJ 3D printer. Pillars were printed with a platen temperature of 90 °C, and walls were printed at 80 °C. Nitrogen flow through the atomizer was set to approximately 25 SCCM and sheath flow was set to approximately 50 SCCM. The substrate size was 25 × 25 × 1.5 mm. A copper substrate is used for the micropillar experiments and a silver substrate for the microwalls. The change in the substrate type was prompted by excessive oxidation of copper that tended to affect the adhesion of the microwalls to the substrate.

### Sintering conditions and curvature measurement

The micropillars and microwalls printed on a substrate were placed on a hot plate for sintering, as shown in Fig. 1b, c. The temperature of the surface of the hot plate (Model HS40, Torrey Pines Scientific, Carlsbad CA) was controlled by a programmed 8-hour cycle. The heating cycle included a ramp to 300 °C at a rate of 3 °C/min; a hold of 5 hours; followed by cooling to room temperature at a rate of 3 °C/min. The AJ printed samples on a substrate were placed/attached on another silver substrate, which was placed on the surface of the hot plate, with thermal paste (boron nitride paste, Slice Engineering, Gainesville FL) applied between each layer. The kinetics of deformation were measured using a high-magnification video camera. The distortions of the micropillars and microwalls were recorded using a camera (Canon EOS T7i, Canon Inc, Tokyo, Japan, with Infini-Probe TS-160 lens from Infini Inc, Centennial CO) as shown in Fig. 1c. Side views of the samples were taken every 15 seconds to create timelapse videos of the walls to track the curvature change.

### Experimental data analysis for wall bending

Side views of the images were recorded at 15-second intervals during sintering and were used for the data analysis. About 22 images at different times were chosen for the curvature analysis of each experiment. Three points representing the wall’s curve were selected from each image in Matlab to estimate the wall curvature at that instant. The walls showed maximum bending at the bottom; therefore, the points selected for curvature consisted of the first one at the base, the second one at the bend near the bottom, and the third one closer to the top. The estimated measurement error was about ± 5%.

### Temperature measurement and analysis

A thermal camera (Model A655, Teledyne FLIR Inc, Wilsonville OR) was used to measure the temperature of the front and back of the microwalls every 10 mins throughout the sintering process. Heat maps were captured as jpg files and individual pixel temperature data was captured as a csv file. Three temperature measurements were taken at every interval (front-back-front), where the temperature of the front of the wall was averaged to mimic concurrent temperature measurements on both sides of the walls. The temperature data/pixels collected as a csv file were analyzed using a program written in Matlab. First, the objects at room temperature (outside of the microwalls) were isolated and removed from the analysis. The microwalls were then isolated from the substrate with a secondary filter in Matlab with a temperature threshold observed from the heatmap. Due to limitations discussed earlier, only qualitative information from these results is meaningful (i.e., the determination of which side of the wall is hotter). This was determined by comparing an average of the captured temperature data for the front and back of each wall.

### Porosity gradient

Focused Ion Beam or FIB (FEI dual beam plasma, FEI Corp, Hillsboro, OR) along with SEM technique was used to cross-section and image the microwalls to examine the porosity gradient through the thickness of the microwalls. The irregular pore shapes of the sintered samples created a significant spread in the data, so we decided to use a different approach to study any potential porosity gradient. A sample which had previously been sintered on a hot plate using the same temperature profile as other experiments was subsequently reheated in an oven at a higher temperature (400 °C) for a longer time (10 hr) to further reduce the porosity. The wall showed no change in shape upon further sintering and had an overall porosity of 9.9% (about half of the original samples). With smaller pores, the analysis becomes much clearer and more meaningful. Nine individual images were taken from the cross-section for this analysis (to accommodate curvature) for each of the five regions through the wall thickness. This data is plotted in Fig. 3e which includes the weighted average and standard deviation across the nine images for each subdivision. The data is weighted by the size of each image as they are not exactly equal.

Porosity data from additional cross-sections using the samples after the original experiments (without additional sintering) are shown in Supplementary Fig. 1a–c (for a total of three walls in this condition, each one 35 µm thick), including a line of best fit. Position ‘1’ is the convex side for each of the walls, while position ‘5’ is the concave side after bending. The porosity data obtained from the FIB images (Supplementary Fig. 1a–c and Fig. 3d–f) was combined and plotted as the porosity difference from the individual sample mean, allowing for comparison between samples with different average porosity. This data accounts for 48 individual cross-section images across four different walls. The average and standard deviation are weighted such that each wall is given equal weight (to remove the effect of each wall requiring a different number of images to accommodate curvature) and individual cross-sections within a wall are weighted by their size as before.

### Experimental thermal analysis

TGA of the as-received nanoparticle ink used in this work was performed (Q50, TA Instruments, New Castle, DE). The ink was heated in an air environment from 25 °C to 500 °C at a ramp rate of 10 °C/min. Additional tests used a ramp rate of 30 °C/min until reaching a dwell temperature, after which the ramp rate of 10 °C/min was used to reach the final temperature of 500 °C. Differential scanning calorimetry or DSC was also performed (Model STA 6000, Perkin Elmer, Waltham, MA) on the as-received nanoparticle ink. The ink was heated to a maximum temperature of 975 °C, again at a ramp rate of 10 °C/min. The test was run twice in succession, first on a sample of 5 µL of ink to capture the effects of solvent evaporation, binder burnout, and sintering and second on the exact same remaining silver to show the results without any of the listed effects.

Additionally, the thermal conductivity of the printed material was measured at various points during the sintering cycle. Above 200 °C, the Wiedemann-Franz Law was utilized. The electrical conductivity of high-aspect-ratio silver lines was measured using the 4 W method^34^, and the cross-section area of the printed and sintered lines was measured using physical profilometry (KLA Tencor P-15 Profilometer, Milpitas, CA). At lower temperatures where lattice contributions dominate over electronic contributions, the Wiedemann-Franz Law is insufficient, so we used an alternative method, frequency-domain thermoreflectance (FDTR)^35^. For this method, a 9 µm thick layer of printed silver ink was coated with a 70 nm thick layer of gold and subjected to an incident laser in a non-contact, pump-probe experiment. In this technique, a phase delay is generated between the intensity modulation of the two lasers due to the material’s thermal responsivity. The phase delay data is fit to a solution of the heat diffusion equation to determine the sample’s thermal conductivity^36^, and the uncertainty is quantified using a Monte Carlo approach^37–39^.

### Mathematical formulation

The rate of change in the potential energy given in Eq. (5) can be written as

11 $$\dot{E}=	 {\int }_{V}\left\{F\dot{\phi }+\frac{\partial Q}{\partial \nabla {\theta }_{S}}\cdot \nabla \dot{\phi }+\frac{\partial Q}{\partial \nabla {\theta }_{m}}\cdot \nabla {\dot{\theta }}_{m}\right\}{dV} \\=	 {\int }_{\partial V}\left\{{{{{{\bf{n}}}}}}\cdot {{{{{\bf{M}}}}}}\dot{\phi }-\alpha {{{{{\bf{n}}}}}}\cdot {{{{{\bf{M}}}}}}{\dot{\theta }}_{m}\right\}d\partial V \\ 	+{\int }_{V}\left\{\left[F-\nabla \cdot {{{{{\bf{M}}}}}}\right]\dot{\phi }+\nabla \cdot (\alpha {{{{{\bf{M}}}}}}){\dot{\theta }}_{m}\right\}{dV}.$$

The dissipative constitutive law connects the power conjugate fields and, for a purely dissipative process, it also provides the governing equations:

12 $$\dot{\phi }=-\mu \left(T\right)\left[F\left(\phi \right)-\nabla \cdot {{{{{\bf{M}}}}}}\right]{{{{{\rm{;}}}}}}\,{\dot{\theta }}_{m}=-\eta \left(\phi \right)\nabla \cdot \left(\alpha {{{{{\bf{M}}}}}}\right){{{{{\boldsymbol{,}}}}}}$$

where the vector field ${{{{{\bf{M}}}}}}=B{l}^{2}\left(\nabla {\theta }_{S}-\alpha \nabla {\theta }_{m}\right)=\partial Q/\partial \nabla {\theta }_{S}$, has dimensions of torque per unit area (i.e., the couple stress). While this may not be obvious in the simplified macro-stress-free theory presented here, the full formulation is a strain gradient theory^40^, albeit of the dissipative type^41^. The boundary conditions in Eq. (7) follow from the requirement that the normal mass flux vanishes at free boundaries: ${{{{{\bf{n}}}}}}\cdot {{{{{\bf{M}}}}}}=0$.

### Dimensional analysis of the one-dimensional problem of wall bending

We consider a slender cross-section of freestanding walls with a temporary lateral temperature gradient. The structure extends in the direction $x$, with a small uniform temperature gradient in the direction $y\in [0,\delta ]$. The sintering is done under nominal temperature ${T}_{0}$, but with maximum temperature variation $\Delta T\ll {T}_{0}$. The temperature $T(y,{t;}{T}_{0},\Delta T)$ is a prescribed function designed to approximate the temperature difference between the sides $y=0,\delta$, obtained by simulations as discussed in Supplementary Note 3. The characteristic length in the process is the wall thickness, $\delta$, and the characteristic time, $1/{\mu }_{0}{F}_{0}$, is the reciprocal of the maximum homogeneous sintering rate, Eq. (8), under nominal temperature. The non-dimensional quantities will be denoted by the overbar, e.g., $\bar{y}=y/\delta$. We consider the problem with linearized kinematics.

The non-dimensional problem is governed by three non-dimensional parameters in Eq. (10) and the prescribed temperature function $\bar{T}(\bar{y},\bar{t};\Delta \bar{T})$. The governing equations are 2nd order, nonlinear PDEs, with boundary and initial conditions:

$$\left\{\begin{array}{c}\frac{\partial \phi }{\partial \bar{t}}=-\bar{\mu }(\bar{T})\left[\bar{F}\left(\phi \right)-\beta \left(\frac{{\partial }^{2}\phi }{\partial {\bar{y}}^{2}}-\alpha \frac{{\partial }^{2}{\theta }_{m}}{\partial {\bar{y}}^{2}}\right)\right]\hfill\\ \frac{\partial {\theta }_{m}}{\partial \bar{t}}=-\bar{\alpha }\beta \bar{\eta }(\phi )\left(\frac{{\partial }^{2}\phi }{\partial {\bar{y}}^{2}}-\alpha \frac{{\partial }^{2}{\theta }_{m}}{\partial {\bar{y}}^{2}}\right)\hfill\end{array}\right\}\bar{y}\in \left[0,1\right],$$

13 $${\left.\frac{\partial \phi }{\partial \bar{y}}\right | }_{\bar{y}=0,1}=\alpha {\left.\frac{\partial {\theta }_{m}}{\partial \bar{y}}\right | }_{\bar{y}=0,1}{{{{{\rm{;}}}}}}\,\phi \left(\bar{y},0\right)={\phi }_{0}{{{{{\rm{;}}}}}}\,{\theta }_{m}\left(\bar{y},0\right)=0.$$

The parameter $\bar{\alpha }$ is the ratio of respective mobilities. When both $\bar{\alpha }\beta \ll 1$ and $\beta \ll 1$, we have homogeneous sintering. The mass transport strain ${\theta }_{m}$ is negligible and the porosity evolves according to Eq. (8). When only $\bar{\alpha }\ll 1$ and $\beta$ is not, the mass transport strain ${\theta }_{m}$ may be negligible (depending on $\bar{\alpha }\beta$), but the gradient in $\phi$ produces the internal sintering pressure, $\beta {\partial }^{2}\phi /\partial {\bar{y}}^{2}$, so that sintering is not homogeneous. Finally, when only $\beta \ll 1$ but $\bar{\alpha }\beta$ is not, the porosity evolves homogeneously, but the mass transport strain ${\theta }_{m}$ is not negligible, nor homogeneous.

### Numerical method

The initial/boundary value problem in Eq. (13) is solved using a second-order finite element space scheme and the forward Euler method for time discretization. The computational domain is discretized with a uniform mesh size $\Delta \bar{y}$ = 10^−2^. The stability of the scheme is ensured by a small timestep. Convergence is checked by comparing solutions for curvature for various mesh sizes and timesteps.

## Supplementary information

Supplementary Information

Description of Additional Supplementary Files

Supplementary Movie 1

Supplementary Movie 2

Supplementary Movie 3

Supplementary Movie 4

## Data Availability

All data needed to evaluate the conclusions in the paper are present in the paper and/or the Supplementary Information. Additional data related to this paper may be requested from the authors.

## Code availability

Code available on request from the authors.

## References

[1] Herzog D (2016). Additive manufacturing of metals. Acta Mater..

[2] Mehrpouya M (2019). The potential of additive manufacturing in the smart factory industrial 4.0: a review. Appl. Sci..

[3] Bandyopadhyay, A. & Bose, S. Additive manufacturing. CRC press (2019).

[4] MacDonald E, Wicker R (2016). Multiprocess 3D printing for increasing component functionality. Science.

[5] Cho, H., et al. Warpage of powder injection molded copper structure. *Met. Mater. Int.* **27**, 1–7 (2019).

[6] Zhang Y, Zhang J (2017). Finite element simulation and experimental validation of distortion and cracking failure phenomena in direct metal laser sintering fabricated component. Addit. Manuf..

[7] Dehghan-Manshadi A (2018). Metal injection moulding of non-spherical titanium powders: processing, microstructure and mechanical properties. J. Manuf. Process..

[8] Dastjerdi AA, Movahhedy MR, Akbari J (2017). Optimization of process parameters for reducing warpage in selected laser sintering of polymer parts. Addit. Manuf..

[9] Schmutzler C (2016). Pre-compensation of warpage for additive manufacturing. J. Mech. Eng. Autom..

[10] Atkin, R.J. & Fox, N. An introduction to the theory of elasticity. Courier Corporation, 2005.

[11] Saleh MS, Hu C, Panat R (2017). Three-dimensional microarchitected materials and devices using nanoparticle assembly by pointwise spatial printing. Sci. Adv..

[12] Kraft T, Riedel H (2004). Numerical simulation of solid state sintering; model and application. J. Eur. Ceram. Soc..

[13] Wakai F (2006). Modeling and simulation of elementary processes in ideal sintering. J. Am. Ceram. Soc..

[14] Al-Qudsi A (2015). Comparison between different numerical models of densification during solid-state sintering of pure aluminium powder. Prod. Eng..

[15] Sahli M (2018). Experimental analysis and numerical simulation of sintered micro-fluidic devices using powder hot embossing process. Int. J. Adv. Manuf. Technol..

[16] Wawrzyk, K., et al. A constitutive model and numerical simulation of sintering processes at macroscopic level. In AIP Conference Proceedings. AIP Publishing LLC (2018).

[17] Martin C, Bouvard D, Shima S (2003). Study of particle rearrangement during powder compaction by the discrete element method. J. Mech. Phys. Solids.

[18] Martin C (2006). Discrete element modeling of metallic powder sintering. Scr. Mater..

[19] Martin CL (2009). Evolution of defects during sintering: discrete element simulations. J. Am. Ceram. Soc..

[20] Balakrishnan A (2010). Effect of particle size in aggregated and agglomerated ceramic powders. Acta Mater..

[21] Hugonnet B (2020). Effect of contact alignment on shrinkage anisotropy during sintering: stereological model, discrete element model and experiments on NdFeB compacts. Mater. Des..

[22] Wang YU (2006). Computer modeling and simulation of solid-state sintering: a phase field approach. Acta Mater..

[23] Zhang R-j (2014). Thermodynamic consistent phase field model for sintering process with multiphase powders. Trans. Nonferrous Met. Soc. China.

[24] Shinagawa K (2014). Simulation of grain growth and sintering process by combined phase-field/discrete-element method. Acta Mater..

[25] Chockalingam K (2016). 2D Phase field modeling of sintering of silver nanoparticles. Comput. Methods Appl. Mech. Eng..

[26] Saleh MS (2018). Polycrystalline micropillars by a novel 3-D printing method and their behavior under compressive loads. Scr. Mater..

[27] Searcy AW (1987). Theory for sintering in temperature gradients: role of long-range, mass transport. J. Am. Ceram. Soc..

[28] Beruto D, Botter R, Searcy AW (1989). Influence of temperature gradients on sintering: experimental tests of a theory. J. Am. Ceram. Soc..

[29] Young RM, Mcpherson R (1989). Temperature-gradient-driven diffusion in rapid-rate sintering. J. Am. Ceram. Soc..

[30] Johnson DL (1990). Comment on “Temperature-gradient-driven diffusion in rapid-rate sintering”. J. Am. Ceram. Soc..

[31] Olevsky EA, Froyen L (2009). Impact of thermal diffusion on densification during SPS. J. Am. Ceram. Soc..

[32] Schottky G (1965). A theory of thermal diffusion based on the lattice dynamics of a linear chain. Phys. Status Solidi (b).

[33] Frost, H. J. & Ashby, M. F. Deformation mechanism maps: the plasticity and creep of metals and ceramics. Pergamon press (1982).

[34] Rahman MT (2016). Structure, electrical characteristics, and high-temperature stability of aerosol jet printed silver nanoparticle films. J. Appl. Phys..

[35] Malen, J. A. et al., Optical measurement of thermal conductivity using fiber aligned frequency domain thermoreflectance. *J. Heat Transfer* **133**, 081601 (2011).

[36] Cahill DG (2004). Analysis of heat flow in layered structures for time-domain thermoreflectance. Rev. Sci. Instrum..

[37] Saha D (2020). Enhancing thermal interface conductance to graphene using Ni–Pd alloy contacts. ACS Appl. Mater. Interfaces.

[38] Wang Z (2022). Nanocrystal ordering enhances thermal transport and mechanics in single-domain colloidal nanocrystal superlattices. Nano Lett..

[39] Christodoulides AD (2021). Signatures of coherent phonon transport in ultralow thermal conductivity two-dimensional ruddlesden–popper phase perovskites. ACS Nano.

[40] Hutchinson J, Fleck N (1997). Strain gradient plasticity. Adv. Appl. Mech..

[41] Fleck N, Willis J (2015). Strain gradient plasticity: energetic or dissipative?. Acta Mech. Sin..

