# Author Correction: Shape distortion in sintering results from nonhomogeneous temperature activating a long-range mass transport

**DOI:** 10.1038/s41467-023-39407-3

**Published:** 2023-06-16

**Authors:** Sandra M. Ritchie, Sasa Kovacevic, Prithviraj Deshmukh, Alexander D. Christodoulides, Jonathan A. Malen, Sinisa Dj. Mesarovic, Rahul P. Panat

**Affiliations:** 1 Department of Mechanical Engineering, Carnegie Mellon University, Pittsburgh, PA USA; 2 School of Mechanical and Materials Engineering, Washington State University, Pullman, WA USA

**Keywords:** Structural properties, Mechanical engineering

Correction to: *Nature Communications* 10.1038/s41467-023-38142-z, published online 09 May 2023

The HTML version of this Article incorrectly omits Supplementary Movie 1, Supplementary Movie 2, Supplementary Movie 3, and Supplementary Movie 4. The Supplementary Movies can be found as Supplementary Information associated with the original article and this Correction, and their descriptions can be found in the file entitled ‘Description of Additional Supplementary Files’.

## Supplementary information

Description of Additional Supplementary Files

Supplementary Movie 1

Supplementary Movie 2

Supplementary Movie 3

Supplementary Movie 4

