# Supplementary material for: Shape distortion in sintering results from nonhomogeneous temperature activating a long-range mass transport
Source: Nat Commun. 2023 May 9;14:2667. doi: 10.1038/s41467-023-38142-z (PMC10169797; doi:10.1038/s41467-023-38142-z)
Supplement: Supplementary file 1 — Supplementary Information [file 41467_2023_38142_MOESM1_ESM.pdf]

Supplementary Information for

Shape distortion in sintering results from nonhomogeneous temperature activating a long-range mass transport

Authors: Sandra M. Ritchie[1,†], Sasa Kovacevic[2,†], Prithviraj Deshmukh[1], Alexander D. Christodoulides[1], Jonathan A. Malen[1], Sinisa Dj. Mesarovic[2,*], Rahul P. Panat[1,*]

Affiliations:

[1]Department of Mechanical Engineering, Carnegie Mellon University; Pittsburgh, USA.

[2]School of Mechanical and Materials Engineering, Washington State University; Pullman, USA.

Corresponding Authors: Rahul Panat (rpanat@andrew.cmu.edu), Sinisa Mesarovic (smesarovic@wsu.edu)

†Equal contribution.

Contents:

Supplementary Notes 1 to 5
Supplementary Table 1
Supplementary Figures 1 to 8

Supplementary Note 1: Additional cross-section analysis

Porosity data obtained for the three original walls is given in Supplementary Fig. 1a-c. Two walls in Supplementary Fig. 1a and 1b show no significant bias towards one side or the other, while the small slope seen is not consistent in sign. The third microwall (Supplementary Fig. 1c) shows higher porosity towards the convex (shorter) side. Data from Supplementary Fig. 1a-1c and Fig. 3d plotted as the porosity difference from the individual sample mean is shown in Supplementary Fig. 1d, allowing for comparison between samples with different average porosities. Based on the line fit, the average values predict less than 1% difference between the far sides of the wall and show slightly higher porosity on the convex side of the bent wall, although this is likely statistically insignificant. Lastly, we estimate the curvature of the bent wall as a function of the porosity difference between the concave and convex sides (e.g., strain difference, $\Delta\varepsilon$ between the two sides is $(\phi_{\mathrm{convex}} - \phi_{\mathrm{concave}}) / 3$, with the curvature being $\Delta\varepsilon / \delta$, where δ is the wall thickness). The predicted curvature based on the observed porosity as a function of the experimentally measured curvature is shown in Supplementary Fig. 1e, which shows no correlation between the observed curvature and the one predicted from measured porosity gradients. Grain size analysis was also performed on the wall which was sintered further; results given in Supplementary Fig. 1f show no significant difference between grains on the convex and concave sides.

Supplementary Note 2: Experimental thermal analysis

Thermogravimetric analysis (TGA) of the as-received silver nanoparticle ink was performed in order to study the temperatures at which solvents and binders are removed from the ink. The TGA plot (Supplementary Fig. 4a) indicates that high volatility solvents in the ink are removed at about 80 °C, with a second phase of evaporation of less volatile solvents before 150 °C. Note, however, that the evaporation events shown in Supplementary Fig. 4a are for an ink sample with higher volume (droplet of several millimeters in diameter) compared to the micron-sized droplets during printing. Furthermore, the TGA data is measured at a fixed heating rate, which does not represent the same thermal history the droplets go through during and after printing. As a basic test of the potential for residual solvent to have an effect on bending, a pair of 20 and 35 μm walls were printed and subjected to an additional drying step (80 °C in a 5 mTorr of vacuum for 24 hr)

prior to the bending experiment. As is evident in Supplementary Fig. 4b, in spite of the additional drying step, both walls still exhibited significant distortion as seen elsewhere in this work.

Three additional TGA data sets were also collected (see Supplementary Fig. 4c-4e for data as a function of time and Supplementary Fig. 4f-4h for the same data, respectively, as a function of temperature) to establish if all the solvent evaporation can happen during printing and if the remaining binder may play a role in wall bending. In these tests, an extended dwell time in the TGA equipment was applied to compare evaporation at a fixed temperature after an initial quick ramp. This test was run at 3 different dwell-temperatures of 80 °C, 70 °C, and 60 °C, to account for the difference in the temperatures for the wall as a function of the wall height during heating. Each sample had 15 μL of ink, enough to print over 150 microwalls, indicating that the actual drying time per wall in this experiment is orders of magnitude less than in our tests. This is true even neglecting the effects of layer-by-layer printing and rapid evaporation of microdroplets having an extremely high surface to volume ratio compared to those studied using TGA. At all studied temperatures (Supplementary Fig. 4c-4h), both phases of solvent evaporation occurred before further temperature increase, showing that an adequate drying time can allow for all measurable solvent evaporation during printing. This is not surprising as solvent evaporation that dries the already printed structure before the subsequent droplets reach the surface is a necessary condition to build 3D structures via aerosol jet printing [1]. In addition to the initial evaporation phases, a third phase of (even smaller) mass loss occurs up to 350 °C. The mass loss beyond the initial solvent evaporation is expected to be from binders.

We also conducted the differential scanning calorimetry (DSC) analysis of the as-received silver nanoparticle ink, as shown in Supplementary Fig. 4i. This graph shows two curves: one for the initial heating of 5 μL of silver ink (dark red curve) and the second for the reheating of silver during an identical heating cycle (light red curve). The latter curve is added to illustrate the heat absorbed without solvents and binders as well as the effects of sintering itself. The initial endothermic peak in Supplementary Fig. 4i shows the effect of solvent evaporation (again, this happens during printing, not sintering). At about 175 °C, the process becomes exothermic with increasing temperature until reaching the melting temperature of silver. This shows the continuous and exothermic nature of the sintering of nanoparticles in contrast to reheating the same amount of solid silver, which is endothermic during the entire cycle. Due to their surface energy, the sintering of nanoparticles leads to heat release, contributing to complex thermal effects within the

sintering structures. As was noted and studied in depth for TGA, the onset of the effects of evaporation, binder burnout, and sintering are likely to be at a slightly lower temperature due to the heating rate of the TGA and DSC equipment being higher than that used in our experiments.

Thermal conductivity of the printed nanoparticle structures was also measured at various stages during the heating cycle used in this work, as shown in Supplementary Fig. 5a and 5b. For temperatures above 200 °C, the Wiedemann-Franz Law was used to determine the thermal conductivity. We measured the electrical conductivity of high-aspect-ratio silver lines printed and subjected to the same thermal profile as that for the microwalls in our study. The conductivity was calculated by measuring the resistance of the wires using the 4W method [2] and calculating the cross section area of the printed and sintered lines using profilometry. It is clear that once the sintering initiates (at temperatures above 150 °C), the thermal conductivity of silver rises rapidly from about 5 ± 2 W/(m·K) at 200 °C to up to 100 W/(m·K) as the sintering progresses. Electrical conductivity was too low to measure with available equipment directly after printing and was on the order of 1 S/m at 150 °C before rising to 6.82×10^5 S/m, with a standard deviation of 2.24×10^5 S/m, at 200 °C (both referencing hot plate temperature).

At lower temperatures, where lattice contributions dominate over electronic contributions, the Wiedemann-Franz Law is insufficient, so an alternative measurement technique, frequency-domain thermoreflectance (FDTR), was used [3]. Based on this measurement technique, the data represents a wide spread with a thermal conductivity of 0.61-7.11 W/(m·K) with a mean conductivity of 2.57 W/(m·K). Although the printed material studied using FDTR was not thermally sintered prior to the test, examination afterwards shows that the incident lasers may have caused local partial sintering of the nanoparticles (i.e., under the sputtered gold layer) as seen in the SEM image with FIB-cut and Energy Dispersive X-ray spectroscopy (EDX) data in Supplementary Fig. 5c-5e. It is thus challenging to estimate the thermal conductivity of the nanoparticle ink with binders. Although the binder composition is proprietary to the ink manufacturer, ethyl cellulose and PVA have been used as binders in colloidal nanoparticle inks [4]. Ethyl cellulose has a thermal conductivity of 0.2 W/(m·K), and PVA has a thermal conductivity of 0.2 W/(m·K), both measured as fully dense solids [5]. Based on the presence of silicon in the EDX data (Supplementary Fig. 5e), a silicone rubber is another likely candidate for the binder material, which would give a thermal conductivity of approximately 0.1-0.2 W/(m·K) [6-8]. The thermal conductivity of silver powders has not been reported. The thermal conductivity

of titanium powder (5 μm size) without binders is reported to be 0.140 W/(m·K) [9]. We estimate that the thermal conductivity rapidly rises from a fraction of W/(m·K) to tens to a hundred W/(m·K) as the sintering progresses. This estimate is used for the simulation in the next section.

Supplementary Note 3: Numerical simulations of heat transfer perturbation with exothermic sintering

We have observed that the initial curvature in walls and needles develops in a short time interval during the early stage of sintering and that it coincides with a visible sintering wave (e.g., Supplementary Movie 4). Therefore, we focus on modeling heat transfer during the early stages of sintering.

Sintering is an exothermal process whereby the internal surface energy of grains is released as heat. We consider the 2D wall problem shown in Supplementary Fig. 6a. The evolution of porosity and temperature is governed by:

$$\begin{aligned} &\dot{\phi} = -\mu(T)F(\phi) \\ &c(\phi)\dot{T} = \nabla\cdot[K(\phi)\nabla T] + \mu(T)F^2(\phi) \end{aligned} \qquad (1)$$

where $F(\phi)$ is the internal sintering pressure (see Eq. (3) of the main manuscript), $c(\phi) = (1-\phi)c_S$ is the heat capacity interpolated linearly to its value for solid metal at vanishing porosity, and thermal conductivity is interpolated with a circular arc, as shown in Supplementary Fig. 6b (see discussion in Supplementary Note 2), so that it is small in the beginning, but rises rapidly as the sintering necks develop. This approximation of thermal conductivity is used as it reflects the overall trends of the experimental results which were measured as a function of temperature rather than porosity. To obtain a distinct start of the sintering, the effective sintering (diffusion) mobility $\mu(T)$ is modified from Eq. (9) to include threshold temperature T_1 where the sintering starts (Supplementary Fig. 6c). The numerical perturbation is introduced through the value of T_1. Physically, one may envision a cluster of smaller particles or a cluster with higher packing density, either of which would begin to sinter. Alternatively, one may consider perturbation in temperature as a result of natural convection around the wall. The term $\mu(T)F^2(\phi)$ is the heat source, where

we assume that the entire internal surface energy (Eq. (3) of the main manuscript) transforms into heat.

The wall is heated at the substrate and the convection heat transfer is assumed on other surfaces. The governing equations are implemented into the COMSOL Multiphysics software. The results are shown in Supplementary Fig. 6d and 6e. Two conclusions can be drawn from the results:

(1) Without a mass transport mechanism, the transient curvature develops but is fully reversed upon further sintering.

(2) The exothermic nature of sintering produces transient thermal instability during the sintering wave propagation. The temperature differences between the two sides of the wall are about 3.5 ºC for the 35 μm wall. As seen in the next section and Fig. 6, these temperature differences are sufficient to produce the observed curvatures.

Supplementary Note 4: Temperature and pressure gradient driven diffusion

Vacancy flux $\mathbf{q}$ is proportional to the gradients of pressure p, vacancy concentration, and temperature T [10, 11]:

$$\mathbf{q} = b\left[3\eta\nabla p - \frac{kTN}{c}\nabla c + \frac{Q_f N}{T}\nabla T\right], \tag{2}$$

where: b is the mobility coefficient, η is the Vegard's law coefficient (of order 1 for vacancies), k is the Boltzmann constant, N is the number of lattice sites per unit volume, and Q_f is the enthalpy of vacancy formation.

We compare the terms arising from pressure and temperature gradients in the order-of-magnitude sense, in the early stages of sintering when the contacts are small. As a representative pressure gradient, we take the ratio of maximum pressure to contact radius. Equilibrated by surface tension (Figure 5c), the well-known contact mechanics formulae give:

$$\left|\nabla p\right| \approx \frac{p_{\max}}{a} = \frac{2E^*}{R}, \tag{3}$$

where E^* is the plane strain modulus and R is the particle radius. For 30 nm silver particles:

$$3\eta \nabla p \approx 2\times 10^{19}\,\frac{\mathrm{N}}{\mathrm{m}^3}\ . \tag{4}$$

For lattice parameter of 0.4 nm (Ag) and $Q_f = 1.05\ \mathrm{eV}/\mathrm{defect} \approx 100\,\mathrm{kJ}/\mathrm{mol}$ [12]:

$NQ_f = 2.6\times 10^9\ \mathrm{J}/\mathrm{m}^3$, so that, with ΔT being the temperature difference across the 30 μm wall:

$$\frac{Q_f N}{T}\nabla T \approx 0.9\times 10^{14}\,\frac{\Delta T}{T}. \tag{5}$$

Thus for $\Delta T/T \approx 10^{-2}$, the temperature gradient contribution to the vacancy flux is seven orders of magnitude smaller than the pressure gradient contribution.

Supplementary Note 5: Parametric analysis of microwall bending

It is instructive to split the parameter $\beta = \overline{\beta}\overline{\lambda}$, where $\overline{\beta} = B/F_0$, $\overline{\lambda} = \ell^2/\delta^2$. The reasons for this separation are practical. In our experiments $\overline{\beta}$ is fixed, while $\overline{\lambda}$ varies. Thus, $\overline{\beta}$ signifies the ratio of elastic stiffness and sintering pressure, while $\overline{\lambda}$ is the geometric parameter. In the parametric analysis shown in Supplementary Fig. 7, we found it most instructive to consider variation in two nondimensional parameters, $\overline{\alpha}$ and $\overline{\beta}$, and two dimensional parameters: the wall thickness δ and the temperature difference ΔT. The reasons for the choice of parameters are:

(i) The effect of the particle size ℓ on the maximum sintering pressure F_0 and characteristic mobilities μ_0 and η_0 (and hence on $\overline{\alpha}$ and $\overline{\beta}$) is hidden in the continuum formulation.

(ii) The changes in the nominal temperature T_0 only affect the time scale of the problem.

The effect of the parameter $\overline{\alpha}$ on the curvature during sintering is shown in Supplementary Fig. 7a. This result is not surprising since higher mass transport mobility η_0 (relative to the sintering mobility μ_0) is bound to produce higher peak and permanent curvatures. Similarly, the effect of temperature difference (Supplementary Fig. 7b) is intuitive: the higher the temperature gradient, the higher the curvature.

The different effects of $\overline{\beta}$ and δ justify our choice of parameters. While $\overline{\beta}$ affects only the ratio of peak to permanent curvature (Supplementary Fig. 7c), the change in the wall thickness δ affects not only the parameter $\overline{\lambda}$, but also the size of the domain over which the temperature difference is imposed (Supplementary Fig. 7d). The numerical results (Supplementary Fig. 8) indicate that the peak curvature scales as $1/\delta$, while the permanent curvature is proportional to $1/\delta^2$.

Mathematically, the effect of $\overline{\beta}$ on the results is easily explained. Large values of β in Eq. (13), practically eliminate $\overline{F}(\phi)$ from the equations so that the rates of ϕ and θ_m are always proportional. Such parallel evolution eliminates recovery of the curvature, whose evolution must be (semi-)monotonic. Physically, large $\overline{\beta}$ represents stiff particle assembly and/or small sintering pressure (small surface energy). Stiff assembly implies fast relaxation by mass transfer so that mass transfer strain occurs (almost) in step with the sintering strain. Small sintering pressure implies a small gradient in sintering pressure and, therefore, (almost) uniform sintering rate once the mass transfer stops.

The key remaining problem is undoubtedly the identification of the mass transfer mechanism. Without full understanding of this mechanism, the prediction of the particle size effect can only be speculative. In addition to affecting the nondimensional parameter $\overline{\lambda} = (\ell/\delta)^2$, the particle size affects the maximum sintering pressure which scales as $F_0 \sim \gamma/\ell$ (where γ is the surface energy). Moreover, the characteristic sintering mobility μ_0 controls the sintering strain, i.e., it is the effective mobility for the whole grain (not the mobility for a specific diffusion mechanism). Therefore, its dependence on the grain size is analogous to the creep strain rate dependence on grain size [13], Depending on the dominant diffusion mechanism: $\mu_0 \sim 1/\ell^2$ or $\mu_0 \sim 1/\ell^3$. The effect of the particle size on the characteristic mass transfer mobility η_0 depends on the dominant mass transfer mechanism. While the biased diffusion mechanism implies that η_0 scales in the same way as μ_0, the particle squeezing mechanism leaves η_0 unaffected by particle size change, leading to a very different predictions. This question requires more sophisticated micro-scale modeling of the relevant mass transfer mechanisms, which is currently underway.

Supplementary Table 1: Model parameters for experiments with 20 μm and 35 μm thick microwalls with corresponding figure numbers in the manuscript.

Exp. #	Wall Thickness (μm)	Exp. Group	Figure # in the manuscript	ΔT (°C)	Model Parameters		
					$\bar{\alpha}$	$\bar{\beta}$	$\bar{\lambda}$
1	20	1	Figure 4a, 6a	3.0*	10	3.75×10^{6}	2.89×10^{-6}
2		2	Figure 4b, 6b				
3		3	Supplementary Figure 2a				
4		4	Supplementary Figure 2b				
5		5	Supplementary Figure 2c				
6		6	Supplementary Figure 2d				
7	35	1	Figure 4a, 6a	3.5*	10	3.75×10^{6}	9.44×10^{-7}
8		2	Figure 4b, 6b				
9		3	Supplementary Figure 2a				
10		4	Supplementary Figure 2b				
11		5	Supplementary Figure 2c				
12		6	Supplementary Figure 2d				

* Value obtained from the heat transfer analysis in Supplementary Note 3.

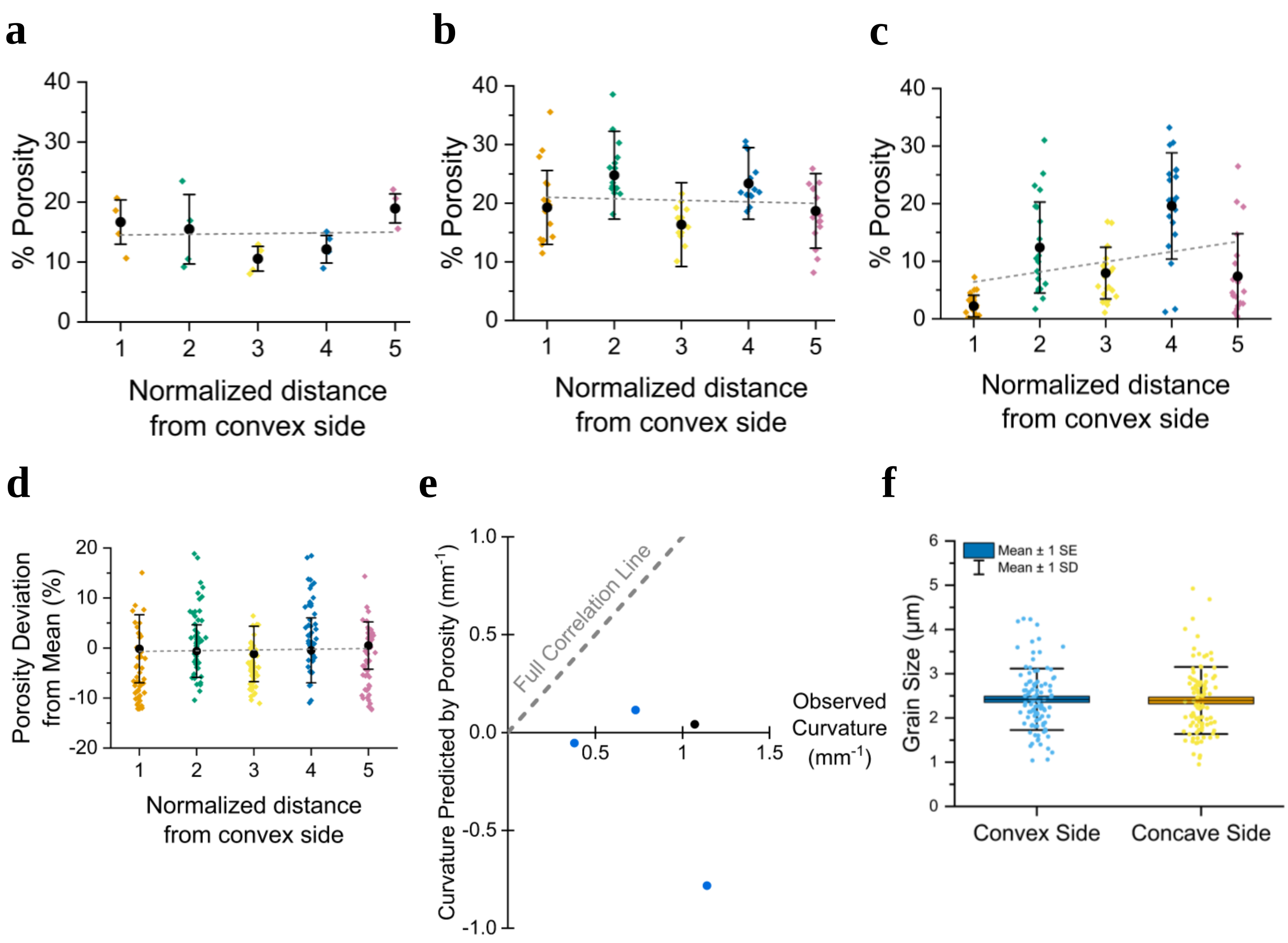

Supplementary Figure 1. Porosity analysis. a-c Porosity as a function of the normalized distance from the convex side for three 3D-printed microwalls subjected to heating per the original experiments described in this work (samples without additional sintering). The standard deviation for the error bar are from multiple FIB sections along the length of each wall. **d** Data from (**a-c**) and Fig. 3d-f plotted as the porosity difference from the individual sample mean, allowing for comparison between samples with different average porosity. Error bars represent standard deviation. **e** Curvature predicted from the measured porosity gradient against the curvature observed in experiments. Positive curvature has the center of curvature on the concave side of the wall. The black dot represents the sample reheated to a higher temperature, shown in Fig. 3d-f. **f** Grain sizes measured from the wall which was further sintered. Data represents 100 randomly chosen grains each from the convex and concave sides. Data shows no significant difference between the two populations using the one-way ANOVA test at the 0.05 level.

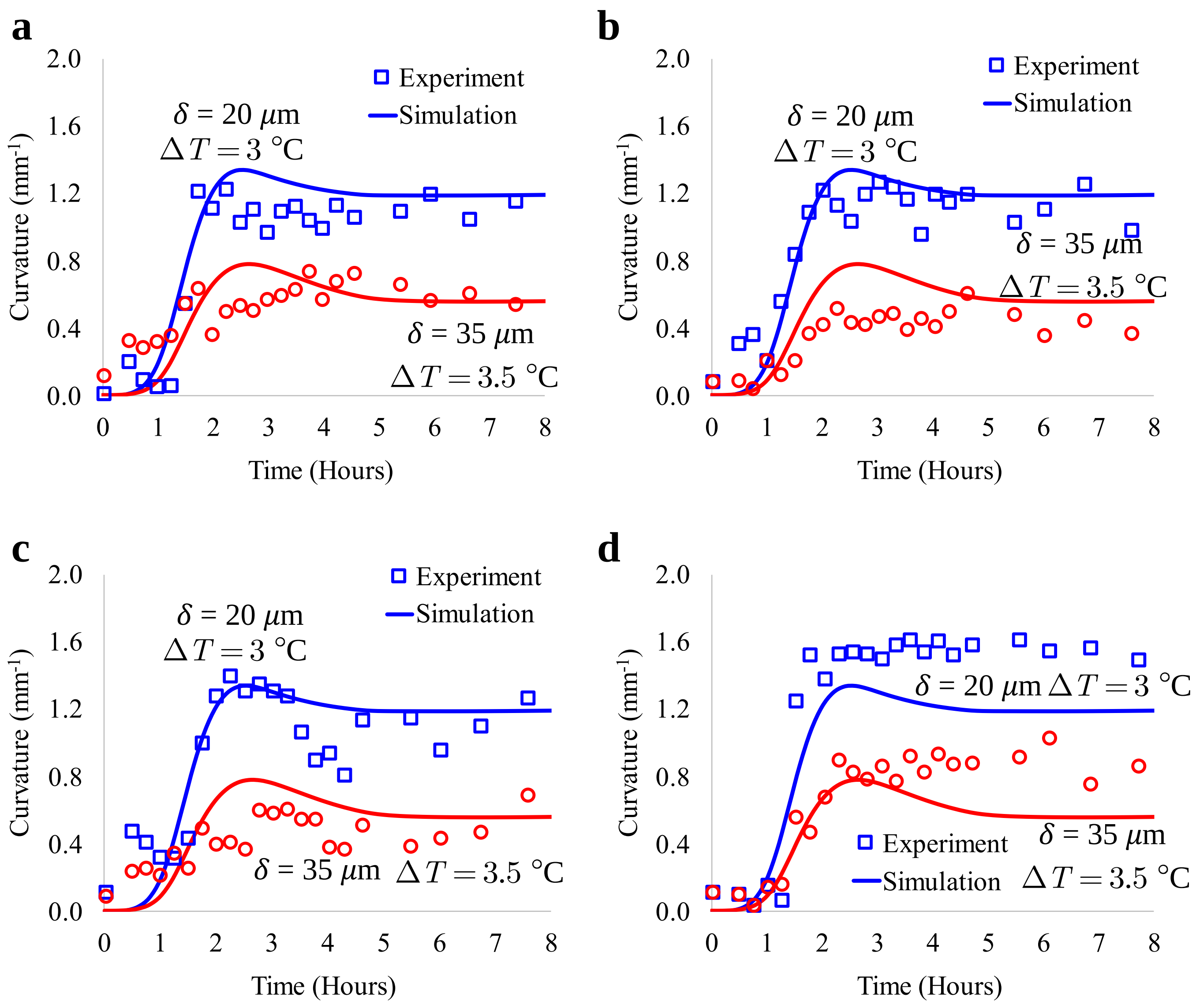

Supplementary Figure 2. Curvature vs heating time. a-d Experimentally observed curvature of 20 and 35 μm thick walls as a function of heating time. Data on additional walls is given in Figure 3a-3b and Fig. 4a-d. Plots comparing the model with the experimental groups three, four, five, and six for walls with 20 and 35 μm thickness. The model parameters used in simulations are given in Supplementary Table 1.

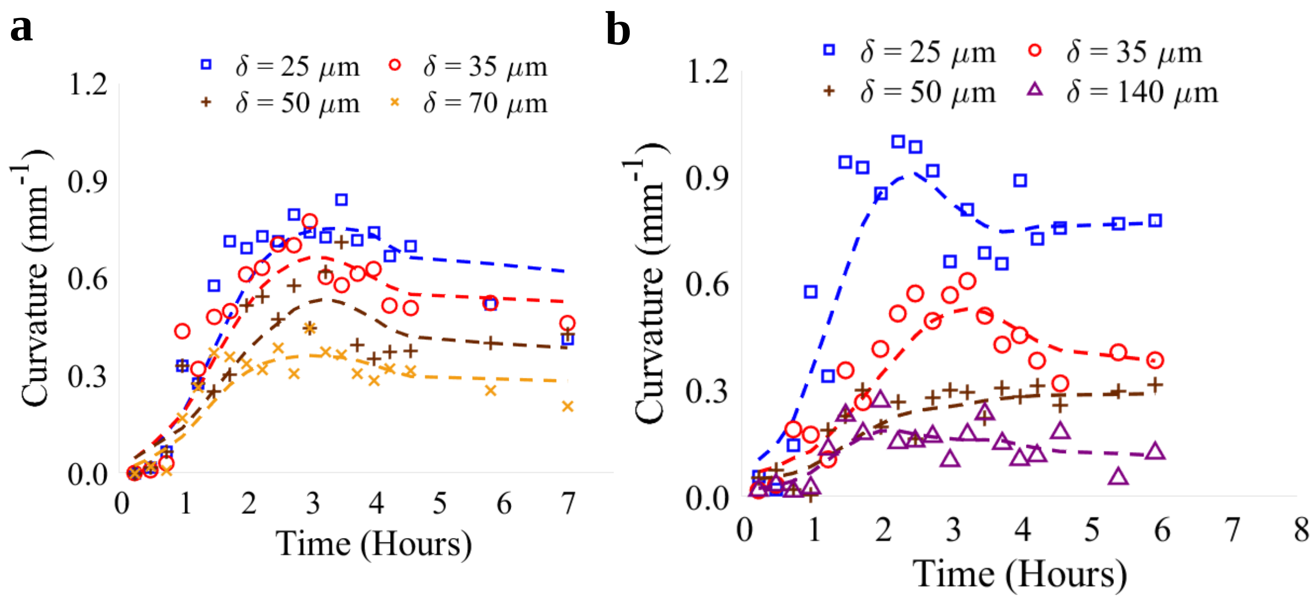

Supplementary Figure 3. Effect of thickness on microwall bending. Experimental measurements for the curvature of freestanding walls for 5-wall geometries. **a, b** Bending of 3D printed wall with thicknesses from 20 to 140 μm. The curvature of the wall with about 140 μm thickness was not visible to the camera as its bending behavior overlapped with that for the 70 μm wall. The inverse is true in (**b**).

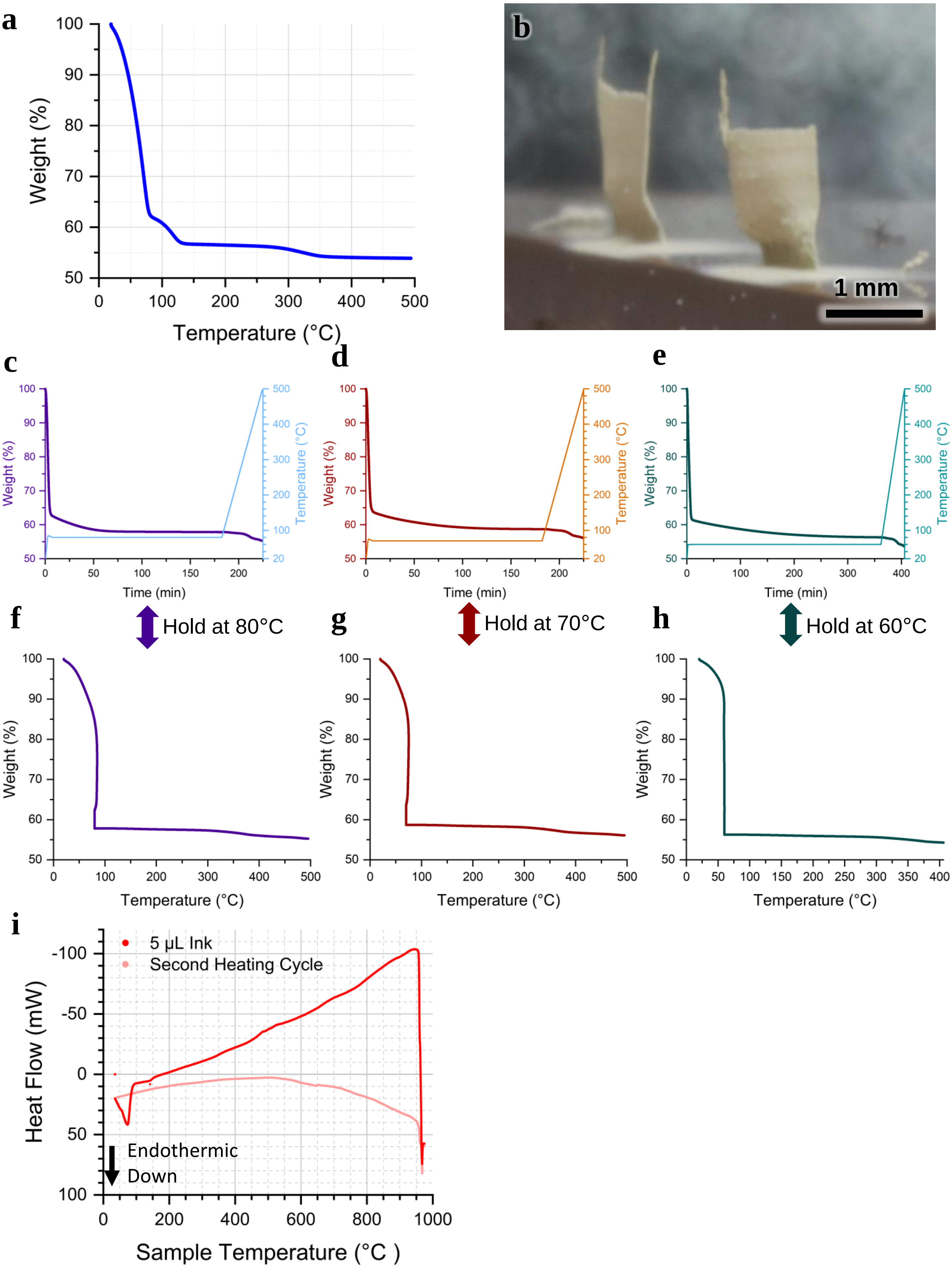

Supplementary Figure 4. DSC and TGA analysis of the silver nanoparticle ink. a Thermogravimetric analysis (TGA) of the ink used in this work. **b** Bent microwalls which prior to sintering had experienced an additional drying step of 80°C heating in a vacuum for 24 hr before sintering. This additional drying step had no apparent effect on distortion behavior. **c-e** TGA of the ink which was held for extended times at the platen printing temperature of 80°C, at the estimated minimum printing temperature of 70°C, and at one temperature below printing conditions of 60°C. All conditions show evaporation of all solvents during the dwell time, even below the used printing conditions. **f-h** Corresponding TGA from (**c-e**) presented as a function of temperature. Note that the second evaporative event seen in (**a**) occurs during the dwell time, even at 60°C. A small mass loss is still apparent between 300-400°C, attributed to residual binder. **i** Differential scanning calorimetry (DSC) of the ink used in this work. The difference between the first and second run data shows the effect of solvent removal, binder burnout, and sintering at different temperatures. The initial endothermic peak shows the effect of solvent evaporation. At about 175°C, the process becomes exothermic with increasing temperature until reaching the melting temperature of silver, in contrast with the entirely endothermic reheating cycle. Note that a faster heating rate is a necessity of the measurement technique, so effects of evaporation, binder burnout, and sintering likely onset at a slightly lower temperature during the sintering process described in this work due to the lower heating rate.

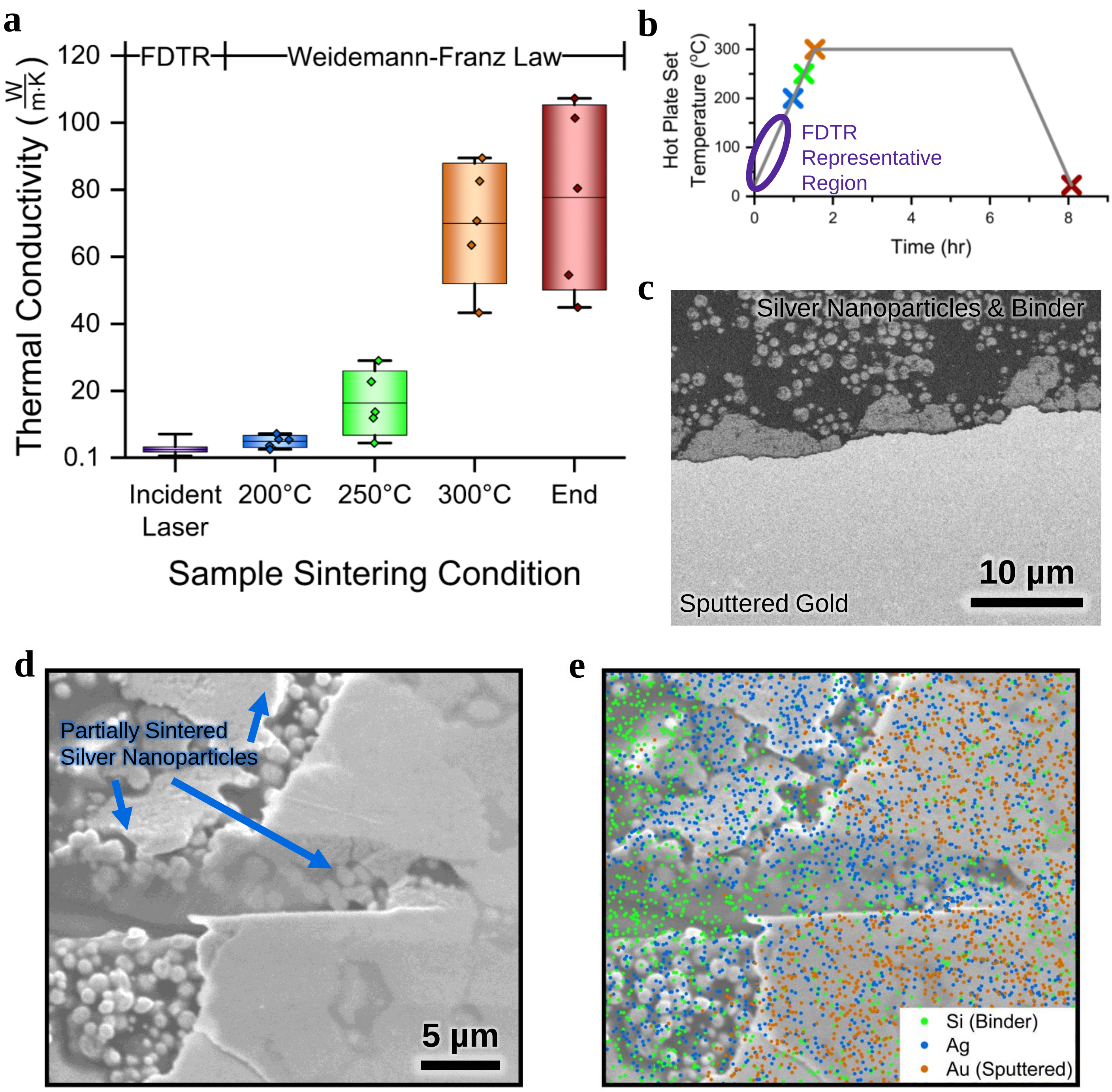

Supplementary Figure 5. Thermal conductivity as a function of heating cycle. a Measured thermal conductivity of the printed ink used in this study (designated by the same-color data points in (**b**). For all temperatures above 200°C, the Wiedemann-Franz law was used to determine the thermal conductivity, meaning that electrical conductivity was measured using four probe testing and physical profilometry in order to calculate the thermal conductivity. At lower temperatures, this relation is insufficient, so an alternative measurement technique was used, frequency-domain thermoreflectance (FDTR). **b** A representative schematic shows the times on the heating cycle for which the thermal conductivity was studied. **c** A backscattered electron micrograph of the sample used for FDTR measurement, including the edge of sputtered gold (brightest color) on top of the printed ink (medium-colored silver and darkly colored binder). **d** FIB cross-section from (**c**) shows that the incident laser caused local partial sintering of the material even without thermally sintering prior to the test. **e** Energy dispersive spectroscopy data from the section given in (**d**) confirming that sintered region is silver. As such, this result shows that the FDTR measures the thermal conductivity after the onset of sintering.

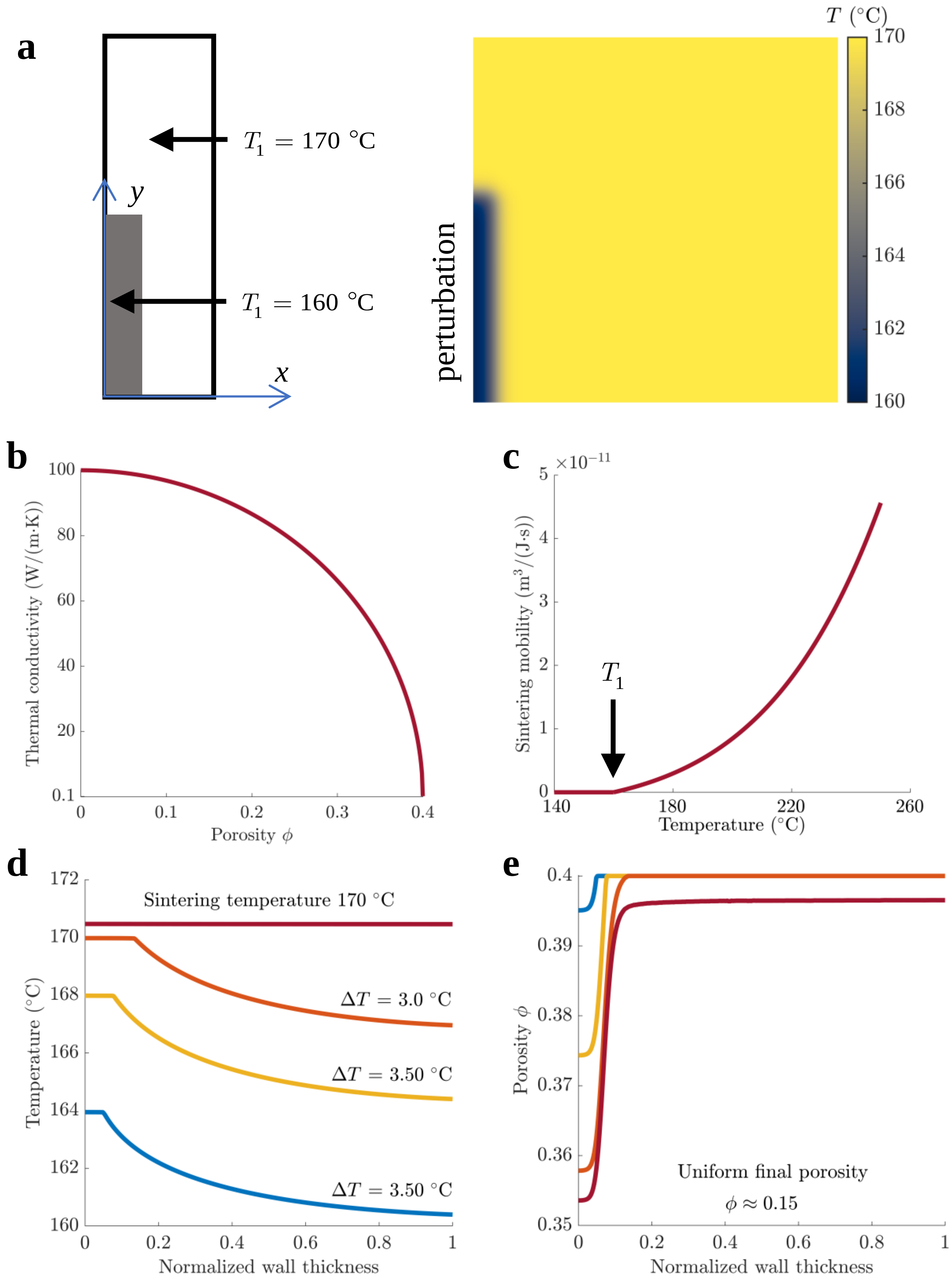

Supplementary Figure 6. Heat transfer simulations for microwalls with thermal perturbations. a Cross section of a wall with indicated perturbation. **b** The dependence of thermal conductivity on porosity. **c** The temperature dependence of mobility. **d** Temperature distribution across the wall at different heating stages in the 35 μm wall. **e** Porosity distribution across the 35 μm wall at different heating stages. The final porosity is uniform.

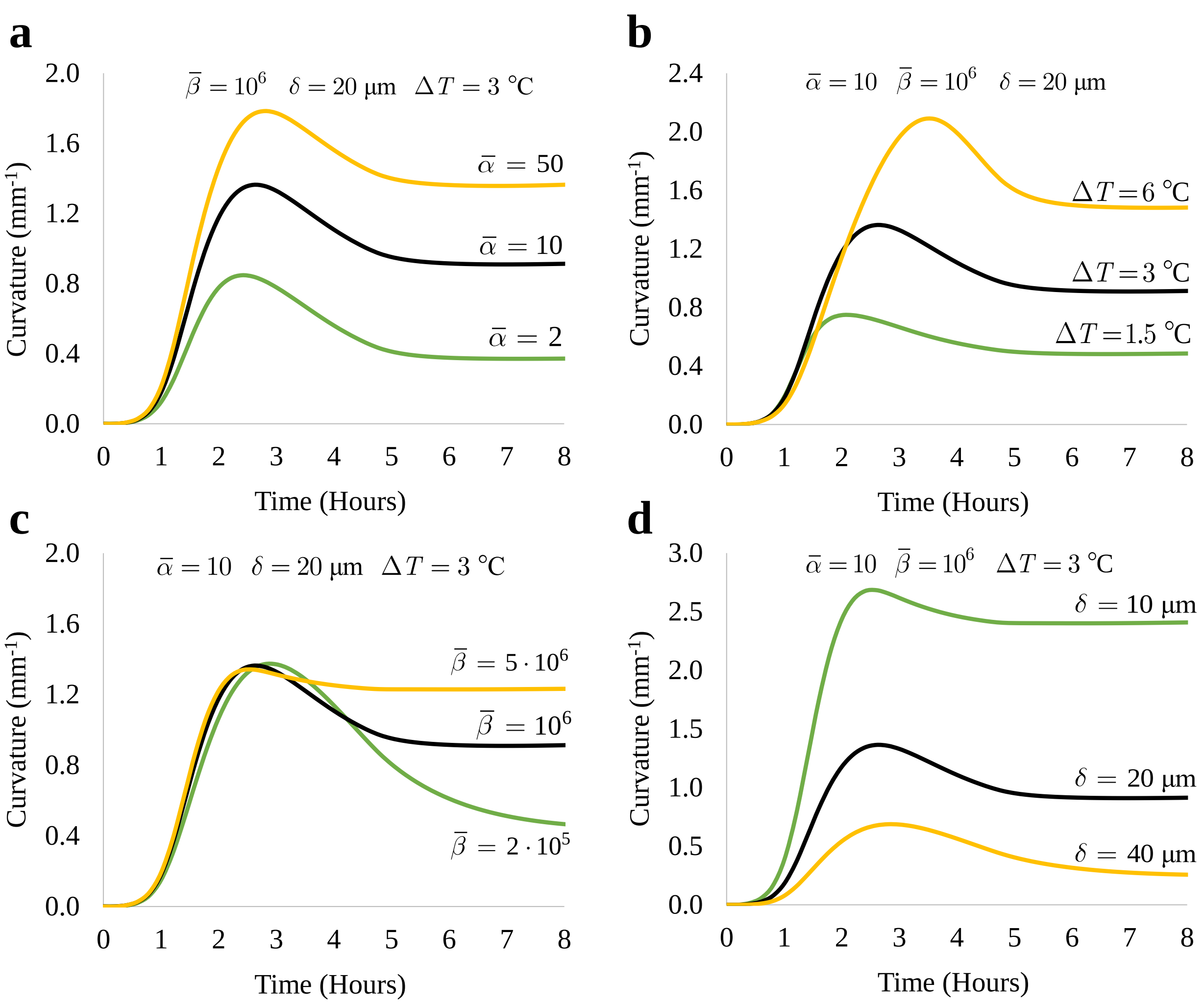

Supplementary Figure 7. Parametric analysis of the bending of microwalls. a-d Parametric analysis using two non-dimensional and two dimensional parameters as discussed in the text. In each figure, one parameter is varied while the others are kept constant.

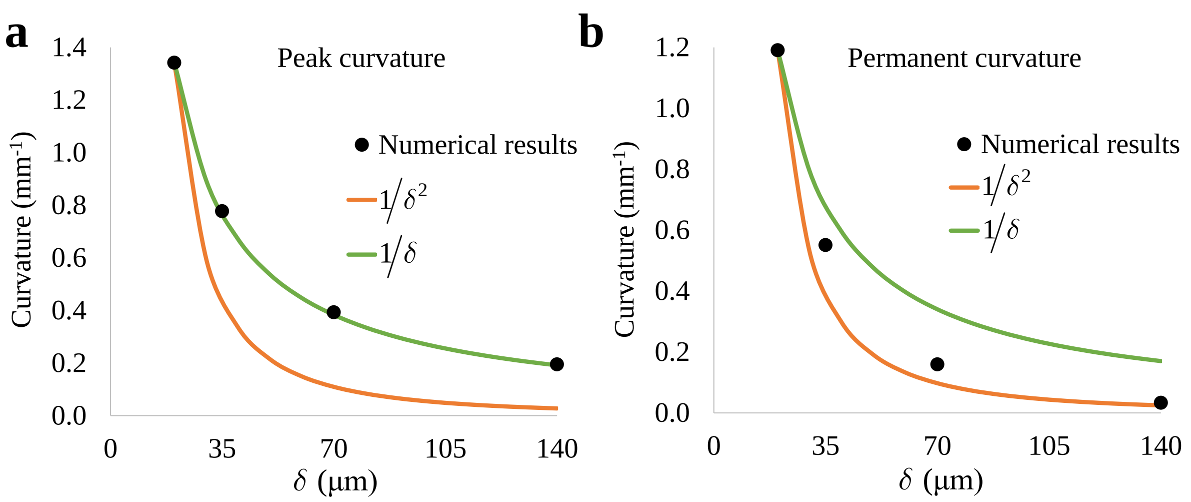

Supplementary Figure 8. Effect of microwall thickness on curvature. Dependence of the peak (**a**) and permanent (**b**) curvature on the wall thickness for $\ell=34$ nm, ΔT=3 °C, $\bar{\alpha}=10$, $\bar{\beta}=3.75\cdot10^6$.

Supplementary References:

1. Saleh, M.S., C. Hu, and R. Panat, *Three-dimensional microarchitected materials and devices using nanoparticle assembly by pointwise spatial printing.* Science advances, 2017. **3**(3): p. e1601986.
2. Rahman, M.T., et al., *Structure, electrical characteristics, and high-temperature stability of aerosol jet printed silver nanoparticle films.* Journal of Applied Physics, 2016. **120**(7): p. 075305.
3. Malen, J.A., et al., *Optical Measurement of Thermal Conductivity Using Fiber Aligned Frequency Domain Thermoreflectance.* Journal of Heat Transfer, 2011. **133**(8).
4. Zeng, M. and Y. Zhang, *Colloidal nanoparticle inks for printing functional devices: emerging trends and future prospects.* Journal of Materials Chemistry A, 2019. **7**(41): p. 23301-23336.
5. Yang, Y., *Thermal Conductivity: Datasheet from · Volume : "Physical Properties of Polymers Handbook" in SpringerMaterials (https://doi.org/10.1007/978-0-387-69002-5_10)*, J.E. Mark, Editor., Springer Science+Business Media, LLC.
6. Yang, D., et al., *High thermal conductive silicone rubber composites constructed by strawberry-structured Al2O3-PCPA-Ag hybrids.* Composites Part a-Applied Science and Manufacturing, 2021. **142**: p. 8.
7. Yin, Z.H., J.H. Guo, and X.H. Jiang, *Significantly improved thermal conductivity of silicone rubber and aligned boron nitride composites by a novel roll-cutting processing method.* Composites Science and Technology, 2021. **209**: p. 8.
8. Mu, Q., S. Feng, and G. Diao, *Thermal conductivity of silicone rubber filled with ZnO.* Polymer Composites, 2007. **28**(2): p. 125-130.
9. Carrion, P.E., et al., *Powder Recycling Effects on the Tensile and Fatigue Behavior of Additively Manufactured Ti-6Al-4V Parts.* JOM, 2019. **71**(3): p. 963-973.
10. Mesarovic, S.D., *Lattice continuum and diffusional creep.* Proceedings of the Royal Society A: Mathematical, Physical and Engineering Sciences, 2016. **472**(2188): p. 20160039.
11. Schottky, G., *A Theory of Thermal Diffusion Based on the Lattice Dynamics of a Linear Chain.* physica status solidi (b), 1965. **8**(1): p. 357-368.
12. Mosig, K., et al., *Investigation of the equilibrium concentration of lattice vacancies in silver and dilute silver-tin alloys with a differential dilatometer.* Journal of Physics: Condensed Matter, 1992. **4**(6): p. 1447.
13. Frost, H.J. and M.F. Ashby, *Deformation mechanism maps: the plasticity and creep of metals and ceramics*. 1982: Pergamon press.
