# Supplementary material for: Shape distortion in sintering results from nonhomogeneous temperature activating a long-range mass transport
Source: Nat Commun. 2023 May 9;14:2667. doi: 10.1038/s41467-023-38142-z (PMC10169797; doi:10.1038/s41467-023-38142-z)
Supplement: Supplementary file 2 — Description of Additional Supplementary Files [file 41467_2023_38142_MOESM2_ESM.docx]

**Description of Additional Supplementary Files**

File Name: Supplementary Movie 1

Description: A video showing a flower-like opening of an AJ-printed 4×4 array of silver micropillars, each with a 60 µm diameter and 4 mm height. The platen was heated to a temperature of 300 °C. The average curvature of the outer micropillars as a function of time is given in Figure 2B. The video is accelerated for clarity with the time indicated in the video.

File Name: Supplementary Movie 2

Description: A video showing the bending of 20 μm and 35 μm thick three dimensional walls of silver nanoparticles as a function of time during sintering. The numbers in the video indicate time.

File Name: Supplementary Movie 3

Description: A video showing the bending of 20 μm and 35 μm thick three dimensional walls of silver nanoparticles as a function of time during sintering. The numbers in the video indicate time.

File Name: Supplementary Movie 4

Description: A video showing the bending of AJ-printed silver nanoparticle walls (1.5×1.5 mm) with increasing thickness from 20 µm to 140 µm. The color change observed in the video corresponds to the sintering front during the heating process. The wall curvatures as a function of time are given in Supplementary Fig. 3.
